# Deconvolving breath alcohol concentration from biosensor measured transdermal alcohol level under uncertainty: a Bayesian approach

**DOI:** 10.3934/mbe.2021335

**Published:** 2021-08-10

**Authors:** Keenan Hawekotte, Susan E. Luczak, I. G. Rosen

**Affiliations:** 1 Department of Mathematics, University of Southern California, Los Angeles, CA 90089, USA; 2 Department of Psychology, University of Southern California, Los Angeles CA 90089, USA

**Keywords:** Bayesian estimation, blood alcohol concentration, breath alcohol concentration, transdermal alcohol concentration, distributed parameter systems, linear semigroup theory, Galerkin methods, posterior consistency, deconvolution

## Abstract

The posterior distribution (PD) of random parameters in a distributed parameter-based population model for biosensor measured transdermal alcohol is estimated. The output of the model is transdermal alcohol concentration (TAC), which, via linear semigroup theory can be expressed as the convolution of blood or breath alcohol concentration (BAC or BrAC) with a filter that depends on the individual participant or subject, the biosensor hardware itself, and environmental conditions, all of which can be considered to be random under the presented framework. The distribution of the input to the model, the BAC or BrAC, is also sequentially estimated. A Bayesian approach is used to estimate the PD of the parameters conditioned on the population sample’s measured BrAC and TAC. We then use the PD for the parameters together with a weak form of the forward random diffusion model to deconvolve an individual subject’s BrAC conditioned on their measured TAC. Priors for the model are obtained from simultaneous temporal population observations of BrAC and TAC via deterministic or statistical methods. The requisite computations require finite dimensional approximation of the underlying state equation, which is achieved through standard finite element (i.e., Galerkin) techniques. The posteriors yield credible regions, which remove the need to calibrate the model to every individual, every sensor, and various environmental conditions. Consistency of the Bayesian estimators and convergence in distribution of the PDs computed based on the finite element model to those based on the underlying infinite dimensional model are established. Results of human subject data-based numerical studies demonstrating the efficacy of the approach are presented and discussed.

## 1. Introduction

Historically, researchers and clinicians interested in tracking alcohol consumption and metabolism in the field would require data from either a drinker’s self-report or from having them use a breath alcohol analyzer. Because both methods require active participation by the subject, the data they produce are often plagued by inaccuracies. Self-report often leads to misrepresentation as (1) subjects may deviate from naturalistic behaviors due to the reporting requirement seeming unnatural, and (2) alcohol directly impairs subjects’ ability to be an active participant [1]. Using a breath alcohol analyzer correctly requires specialized training and can produce erroneous measurements due to mouth alcohol and/or a reading based on a shallow breath by the subject. Dating back to the 1930’s, ethanol, the type of alcohol in alcoholic beverages, has been known to be excreted from the human body through the skin [2–5]. This is due to the fact that water and ethanol are highly miscible [6] and the ethanol finds its way into all of the water in the body. More recently, this observation paved the way for the development of a device to measure the amount of alcohol excreted transdermally through the skin [7–9]. The benefits derived from such a device include the availability of near continuous measurements and the ability to collect them passively (i.e., without the active participation of the subject). This gives researchers and clinicians the potential to continuously observe naturalistic drinking behavior and patterns. There is also the possibility of making these devices available on the consumer market (e.g., wearable body system monitoring technology like Fitbits, Apple watches, etc.). In addition, the ideas we discuss here may also be applicable to the monitoring of other substances once the appropriate sensor hardware has been developed.

The challenge in using transdermal alcohol sensors is that they provide transdermal alcohol concentration (TAC), whereas alcohol researchers and clinicians have always based their studies and treatments on measurements of breath alcohol concentration (BrAC) and blood alcohol concentration (BAC). Thus, a means to reliably and accurately convert TAC to BrAC or BAC would be desirable. At levels up to approximately 0.08 (see, for example, [13, 14]) BrAC correlates well with BAC via a simple linear relationship based on an empirical relationship known as Henry’s law [10, 11]: BAC = *ρ*_*B/Br*_ × BrAC, where the constant *ρ*_*B/Br*_ is known as the partition coefficient of ethanol in blood and breath.

More generally, according to Henry’s law, when a liquid is in contact with a gas, the concentrations, *C*_*L*_ and *C*_*G*_, of a compound present in both the liquid and the gas will come to equilibrium according to the linear relationship *C*_*L*_ = *ρ*_*L/G*_*C*_*G*_, where the empirical determined constant *ρ*_*L/G*_ is known as the partition coefficient for the that compound in that liquid and gas. Not surprisingly, the partition coefficient, *ρ*_*L/G*_, is temperature dependent and of course its actual value will vary depending on the choice of units for *C*_*L*_ and *C*_*G*_. It has been shown (see, for example, [12]) that at 34°C, the partition coefficient for ethanol in blood and air is *ρ*_*B/A*_ = 2157 ± 9.6 for men and *ρ*_*B/A*_ = 2195 ± 10.9 for women, at 37°C, the partition coefficient for ethanol in blood and air is *ρ*_*B/A*_ = 1783 ± 8.1 for men and *ρ*_*B/A*_ = 1830 ± 7.8 for women. Using a regression model, Jones [12] found that at 37°C the partition coefficient for ethanol in water and air is *ρ*_*W/A*_ = 2133, in blood and air is *ρ*_*B/A*_ = 1756, and between plasma (all of the components of blood with the exception of the oxygen carrying red blood cells) and air is *ρ*_*P/A*_ = 2022. All of these values are for the case when the concentration of ethanol in air is given in units of grams per liter, and in water, blood, or plasma in units of grams per deciliter. We note that it is generally accepted that a BrAC reading of 0.08 percent alcohol corresponds to .008 grams of ethanol per 210 liters of breath and a BAC of 0.08 grams of ethanol per 100 milliliters (equal to 1 deciliter (dL) or 0.1 liters (L)) of blood.

Unfortunately, however, the correlation between TAC and BrAC/BAC, on the other hand, can vary due to a number of confounding factors. These factors include, but are not limited to, stable features of the skin like its thickness, tortuosity, and porosity, particularly as they apply to the epidermal layer of the skin, which does not have an active blood supply. Environmental factors such as ambient temperature and humidity can also affect both perspiration and vasodilation, and can thus alter skin conductance, blood flow, the amount of alcohol passing below the skin in the blood, and the amount and rate of alcohol diffusing through the skin. One would also expect there to be manufacturing and operational variations among different TAC sensors.

Earlier attempts to investigate the relationship between TAC and BrAC/BAC have used deterministic models [15–21]. Some utilized regression-based models [16], whereas others utilized first principles physics-based models that on occasion included modeling the transport of alcohol all the way from ingestion to excretion through the skin [22,23]. In our group’s initial efforts, we modeled the transport of alcohol from the blood in the dermal layer through the epidermal layer and its eventual measurement by the sensor using a one-dimensional diffusion equation [15, 21]. The parameters in the diffusion equation model then had to be fit or tuned (i.e., calibrated) to each individual subject, the environmental conditions, and the device through the use of simultaneous BrAC/TAC training data collected in the laboratory or clinic through a procedure known as an alcohol challenge. Once the model was fit, it could then be used to deconvolve BrAC from TAC collected in the field. This two-pass approach and the related studies were relatively successful [15, 19–21, 24, 25]. However, this calibration procedure is quite burdensome to researchers, clinicians, subjects and patients, and because the models were fit to a single uni-modal drinking episode, unaccounted for variation and uncertainty in the relationship between BrAC and TAC frequently arose, making it difficult to accurately convert TAC collected in the field to BrAC [26, 27].

More recently, to eliminate the need for calibration, deconvolution of BrAC from TAC was effected using population models fit to BrAC/TAC training data from drinking episodes across a cohort of subjects, devices, and environmental conditions [24,25,28]. These population models took the form of the deterministic transport models but now the parameters appearing in the model equations were considered to be random. Then in fitting the models, instead of estimating the actual values of these parameters, it was their joint distributions that were estimated. Once the models were fit, they could be used to deconvolve an estimate of the BrAC input, and by making use of the distribution of the population parameters, conservative error bands could also be generated which quantified the uncertainty in the estimated BrAC [24, 25]. The results in these studies were based on a naive pooled data statistical model and a non-linear least squares estimator.

In this paper, we seek to build on the approach described in the previous paragraph by now using a Bayesian approach to account for the underlying uncertainty and variation in the alcohol diffusion and measurement process. We obtain posterior distributions for the transport model parameters conditioned on the training BrAC/TAC data and regularized by prior distributions based on deterministic fits. Being Bayesian based, our approach yields credible sets for the estimated parameters and what we shall refer to as *conservative credible or error bands* for the deconvolved estimated BrAC. What is meant by the term conservative credible band will be made precise later.

An outline of the remainder of the paper is as follows. In the next section of the paper we provide a description of our method including a derivation of a new abstract parabolic hybrid PDE/ODE model for the transdermal transport of alcohol through he epidermal layer of the skin and its capture in the vapor collection bay of the sensor. Then using linear semigroup theory we obtain an input/output model in the form of a discrete time convolution. A discussion of finite dimensional approximation and convergence issues related to the use of our model to carry out the requisite computations is also included. Then in the results section of the paper we first construct our Bayesian estimator and present two theoretical results related to it: convergence of the finite dimensional approximation and consistency. We then show how our population model based on Bayesian estimates for the random parameters can be used as part of a deconvolution scheme that yields estimated BrAC curves and conservative credible or error bands from a biosensor provided TAC signal. In this section we also present and discuss a sample of our numerical findings demonstrating the efficacy of our approach. Our numerical studies were based on human subject data collected in the Luczak laboratory in the Department of Psychology at USC. A final section contains some discussion of our theoretical and numerical results along with a few concluding remarks and avenues for possible future research.

## 2. Methods

### 2.1. A distributed parameter model for the transdermal transport of alcohol

As in [21] and [24], and making use of an idea recently introduced in [28], we model the alcohol biosensor problem described in Section 1 using a one dimensional diffusion equation to describe alcohol transport through the epidermal layer of the skin coupled with an inflow/outflow compartment model to describe the perspiration vapor collection chamber of the TAC biosensor.

The epidermal layer of the skin sits atop the dermal layer. The dermal layer has an active blood supply while the epidermal layer does not. The latter consists of both dead (the stratum corneum layer which is closest to the surface) and living (the deeper layers closer to the dermal layer) cells surrounded by interstitial fluid. Not having an active blood supply, the cells in the epidermal layer obtain nourishment primarily from O_2_ that diffuses in from the environment beyond the skin.

**Figure 1.**
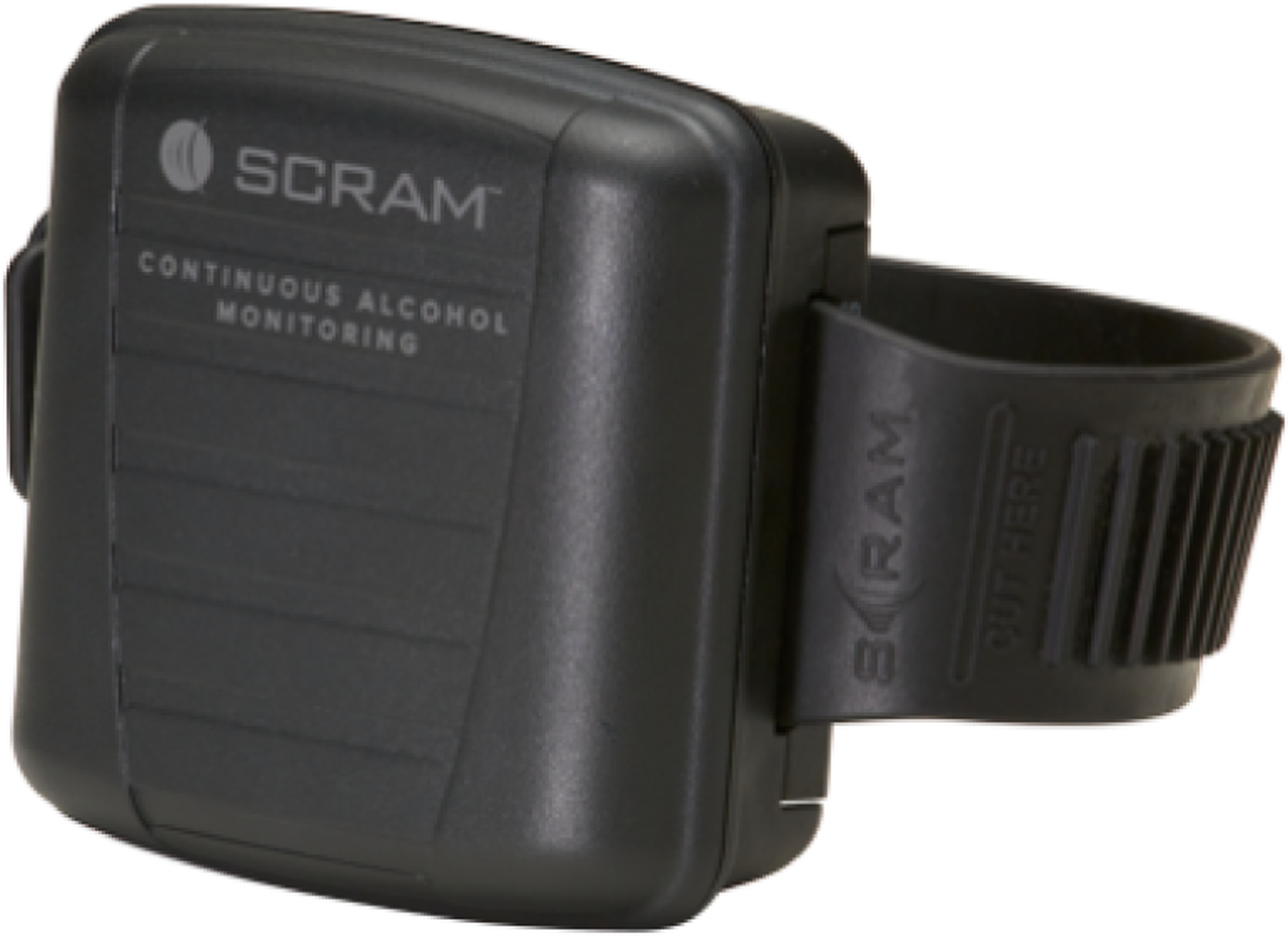
SCRAM Systems transdermal continuous alcohol monitoring device.

The SCRAM TAC biosensor (see fig. 1 in section 3.4 below) has a perspiration vapor collection chamber on the bottom of the sensor that sits atop, and is in direct contact with, the stratum corneum layer of the skin’s epidermal layer. Perspiration in vapor form collects in the chamber. A small pump extracts a sample of the vapor from the collection chamber approximately once every 30 minutes. This sample is then electro-chemically analyzed based on an oxidation-reduction (redox) reaction in much the same way that a fuel cell produces a current (and heat and water) from hydrogen and oxygen. In the TAC sensor, ethanol molecules in the sample are oxidized producing electrons in the form of an electrical current. This current is converted into the TAC measurement based on an a priori bench calibration.

To make this more precise, we let Λ denote the thickness of the epidermal layer (units: cm) of the skin at the location of the sensor and let *η* denote the depth in the epidermal layer (units: cm), 0 ≤ *η* ≤ Λ, *η* = 0 denoting the skin surface and *η* = Λ denoting the boundary between the epidermal and dermal layers. Let *t* denote time (units: hrs) and let *x*(*t*, *η*) denote the concentration of ethanol at time *t* and depth *η* in the epidermal layer (units: grams per milliliter of interstitial fluid). Let $\tilde{w}(t)$ denote the concentration of ethanol in the TAC sensor collection chamber at time *t* (units: grams per milliliter of air), and let *u*(*t*) denote the BrAC at time *t* (units: grams per milliliter of air). Let *y*(*t*) denote the TAC at time *t* (units: grams per milliliter of air), and let ${\tilde{w}}_{0}$ (units: grams per milliliter of air) and *φ*_0_ (units: grams per milliliter of interstitial fluid) denote the initial conditions for $\tilde{w}$ and *x*, respectively. We will assume that there is no ethanol in either the epidermal layer or the TAC biosensor collection chamber at time *t* = 0 so ${\tilde{w}}_{0}=0$ and *φ*_0_ = 0. Let *T* denote the duration of the drinking episode (units: hrs). Then, With these definitions, our model takes the form

(2.1) $$\frac{\partial x}{\partial t}(t,\eta )=\alpha \frac{\partial^{2}x}{\partial \eta^{2}}(t,\eta ),\;0<\eta <\Lambda ,\;0<t<T,\;\;\;\;\;\;\;\frac{d\tilde{w}}{dt}(t)=\frac{\gamma \alpha }{\rho_{P/A}}\frac{\partial x}{\partial \eta }(t,0)-\delta \tilde{w}(t),\;0<t<T,\;\;\;\;\;x(t,0)=\rho_{P/A}\tilde{w}(t),\;0<t<T\;\alpha \frac{\partial x}{\partial \eta }(t,\Lambda )=\beta \rho_{P/A}u(t),\;0<t<T,\;\;\;\;\;\;\;\tilde{w}(0)={\tilde{w}}_{0},\;x(0,\eta )=\varphi_{0}(\eta ),\;0<\eta <\Lambda ,\;\;\;\;\;\;\;\;y(t)=\theta \tilde{w}(t),\;0<t<T,$$

where *α* > 0 denotes the *effective* diffusivity of ethanol in the interstitial fluid in the epidermal layer (units: cm^2^/hr), *β* > 0 denotes the *effective* linear flow rate at which capillary blood plasma from the dermal layer replenishes the interstitial fluid in the epidermal layer (units: cm/hr), and *ρ*_*P/A*_ denotes the partition coefficient for ethanol in plasma and air with respect to the concentration units of grams per milliliter of plasma and grams per milliliter of air at 37°C (normal body temperature).

In modeling the TAC collection chamber, we assume that the inflow of ethanol is proportional to the flux out of (i.e., from right to left) the epidermal layer at the surface of the skin (i.e., at *η* = 0), $\alpha \frac{\partial x}{\partial \eta }(t,0)$, with constant of proportionality *γ* (units: cm^−1^), and the outflow is simply proportional to the concentration of ethanol in the collection chamber (i.e., a simple linear model) with constant of proportionality *δ* (units: hr^−1^. Finally, the output gain, *θ*, represents the bench calibration factor for the TAC sensor that converts the concentration or ethanol in the collection chamber into a TAC (units: dimensionless).

Since the thickness of the epidermal layer, Λ, is in general difficult to measure and can be mathematically difficult to estimate computationally due to it determining the spatial domain of the diffusion equation, it is desirable to transform the system eq. (2.1) to a domain of fixed length, Λ = 1. We make the standard change of variable $\eta \mapsto \frac{\eta }{\Lambda }$ thus rendering *η* dimensionless. For *t* ≥ 0, We also set $w(t)=\rho_{P/A}\tilde{w}(t)$. Then, recalling our assumption of zero initial conditions, the following hybrid ordinary-partial differential equation input/output system results

(2.2) $$\frac{\partial x}{\partial t}(t,\eta )=q_{1}\frac{\partial^{2}x}{\partial \eta^{2}}(t,\eta ),\;0<\eta <1,\;t>0,\;\;\;\;\;\;\;\frac{dw}{dt}(t)=q_{3}\frac{\partial x}{\partial \eta }(t,0)-q_{4}w(t),\;t>0,\;\;\;\;\;\;x(t,0)=w(t),\;t>0\;q_{1}\frac{\partial x}{\partial \eta }(t,1)=q_{2}u(t),\;t>0,\;\;\;\;\;\;\;\;\;\;\;w(0)=0,\;x(0,\eta )=0,\;0<\eta <1,\;\;\;\;\;\;\;\;\;\;\;y(t)=w(t),\;t\geq 0,$$

where $q_{1}=\frac{\alpha }{\Lambda^{2}}$, $q_{2}=\frac{\theta \beta \rho p/A}{\Lambda }$, $q_{3}=\frac{\gamma \alpha }{\Lambda }$, and *q*_4_ = *δ*. We note that since the only observable and observed quantities are BrAC, *u*, and TAC, *y*, the physiological interpretations of the variables and parameters in between that define our model in the form of an input/output map from BrAC to TAC are of little interest to us. Although we have relied on first principles modeling to derive the system of equations given in eq. (2.2), our motivation was not to gain a deeper understanding of the transdermal transport of ethanol. Rather, it was to be able to keep the dimension of the space of unknown parameters as low as possible by capturing the underlying physics and physiology of the transport process, albeit in a greatly simplified form. Indeed, our primary objective here is to first fit the parameters (or, more precisely, their distributions) in the model to observed input/output BrAC/TAC training pairs and to then use the resulting population model to obtain an estimate for the BrAC and associated error bars corresponding to a given TAC signal collected in the field from a member of the cohort or population that provided the data which was used to train the model.

Let ***q*** = [*q*_1_, *q*_2_]^*T*^ denote the unknown, un-measurable, and, in general, subject-dependent physiological parameters. The parameters *q*_3_ and *q*_4_ are device (i.e., hardware) dependent parameters and as such, we consider them to be bench-measurable empirically in the lab. We do note however, with simple changes of variable, the theory and methods we develop below apply, and their distributions could also be estimated along with those of *q*_1_ and *q*_2_ with the same techniques we use here to estimate the distributions of *q*_1_ and *q*_2_. In addition, *q*_3_ and *q*_4_ could also be estimated using a deterministic scheme such as a regularized nonlinear least squares approach. For clarity and ease of exposition, we will focus our attention here on the development of a population model for a cohort of subjects by estimating the distribution of the un-measurable physiological parameters *q*_1_ and *q*_2_.

We consider this system on a finite-time horizon, 0 ≤ *T* < ∞, and we assume zero-order hold input, *u*(*t*) = *u*_*k*_, *t* ∈ [*kτ*, (*k* + 1)*τ*), *k* = 0, 1, 2, ..., where *τ* denotes the sampling time of the biosensor. We set *x*_*k*_ = *x*_*k*_(*η*) = *x*(*kτ*, *η*), *w*_*k*_ = *w*(*kτ*), and *y*_*k*_ = *y*(*kτ*), *k* = 0, 1, . . . , *K*, where we assume *T* = *Kτ*. For *k* = 0, 1, 2, ... we consider the system eq. (2.2) on the interval [*kτ*, (*k* + 1)*τ*] and make the change of variable: *v*(*t*, *η*) = *x*(*t*, *η*) − *ξ*(*η*)*u*_*k*_ where $\xi (\eta )=\frac{q_{2}}{q_{1}}\eta$. It is then easily verified that *w* and *v* satisfy the following hybrid system

(2.3) $$\frac{\partial v}{\partial t}(t,\eta )=q_{1}\frac{\partial^{2}v}{\partial \eta^{2}}(t,\eta ),\;0<\eta <1,\;k\tau <t<(k+1)\tau ,\;\;\;\;\frac{dw}{dt}(t)=q_{3}\frac{\partial v}{\partial \eta }(t,0)-q_{4}w(t)+\frac{q_{3}q_{2}}{q_{1}}u_{k},\;k\tau <t<(k+1)\tau ,\;\;\;\;v(t,0)=w(t),\;k\tau <t<(k+1)\tau ,\;q_{1}\frac{\partial v}{\partial \eta }(t,1)=0,\;k\tau <t<(k+1)\tau ,$$

with initial conditions *v*(*kτ*, ·) = *x*(*kτ*, ·) − *ξ*(·)*u*_*k*_ = *x*_*k*_ − *ξu*_*k*_ and *w*(*kτ*) = *w*_*k*_ on [0, 1].

We then use linear semigroup theory to rewrite the system eq. (2.3) in state space form in an appropriately chosen Hilbert space and subsequently obtain a discrete time evolution system for (*w*_*k*_, *x*_*k*_), *k* = 0, 1, 2, ....*K* which is equivalent to eq. (2.2). Let *Q* denote a compact subset of the positive orthant of ${\mathbb{R}}^{4}$, and for ***q*** = [*q*_1_, *q*_2_, *q*_3_, *q*_4_]^*T*^ ∈ *Q* we define the Hilbert spaces

(2.4) $$H_{q}=\mathbb{R}\times L_{2}(0,1)\;\;\text{and}\;\;V=\left\{\left(\theta ,\psi )\in H_{q}:\psi \in H^{1}(0,1),\theta =\psi (0\right)\right\}$$

with respective corresponding inner products ${\langle \left(\theta_{1},\psi_{1}\right),\left(\theta_{2},\psi_{2}\right)\rangle }_{q}=\left(q_{1}/q_{3}\right)\theta_{1}\theta_{2}+\int_{0}^{1}\psi_{1}(\eta )\psi_{2}(\eta )d\eta$ and ${\langle \left(\psi_{1}(0),\psi_{1}\right),\left(\psi_{2}(0),\psi_{2}\right)\rangle }_{V}=\psi_{1}(0)\psi_{2}(0)+\int_{0}^{1}\psi_{1}^{\prime }(\eta )\psi_{2}^{\prime }(\eta )d\eta$, and respective norms |·|_***q***_, and ∥·∥_*V*_. Note that the Sobolev Embedding Theorem [29] yields that the norm induced by the *V* inner product is equivalent to the standard *H*^1^ norm on *V*. It is not difficult to show that *V* is densely and continuously embedded in *H*_***q***_ and that we have the Gelfand triple of dense and continuous embeddings *V* ↪ *H*_*q*_ ↪ V*.

Then based on the weak formulation of the system eq. (2.3), for each ***q*** ∈ *Q* define the bilinear form a(q,⋅,⋅):V×V→ℝ by $a(q,\hat{\varphi },\hat{\psi })=\left(q_{1}q_{4}/q_{3}\right)\varphi (0)\psi (0)+q_{1}\int_{0}^{1}\varphi^{\prime }(\eta )\psi^{\prime }(\eta )d\eta$ for $\hat{\varphi }$, $\hat{\psi }\in V$, where $\hat{\varphi }=(\varphi (0),\varphi )$, $\hat{\psi }=(\psi (0),\psi )$, and ***q*** = [*q*_1_, *q*_2_, *q*_3_, *q*_4_]^*T*^ ∈ *Q*. Standard arguments can be used to argue that the form *a*(***q***, ·, ·) satisfies the following three properties.

1. **Boundedness** There exists a constant *α*_0_ > 0 such that $\left|a\left(q,{\hat{\psi }}_{1},{\hat{\psi }}_{2}\right)\right|\leq \alpha_{0}{\left\|{\hat{\psi }}_{1}\right\|}_{V}{\left\|{\hat{\psi }}_{2}\right\|}_{V}$, ${\hat{\psi }}_{1}$, ${\hat{\psi }}_{2}\in V$,
2. **Coercivity** There exists constants $\lambda_{0}\in \mathbb{R}$ and *μ*_0_ > 0 such that $a(q,\hat{\psi },\hat{\psi })+\lambda_{0}|\hat{\psi }{|}_{q}^{2}\geq \mu_{0}\|\hat{\psi }{\|}_{V}^{2}$, $\hat{\psi }\in V$.
3. **Continuity** For all ${\hat{\psi }}_{1}$, ${\hat{\psi }}_{2}\in V$, we have that $q\mapsto a\left(q,{\hat{\psi }}_{1},{\hat{\psi }}_{2}\right)$ is a continuous mapping from *Q* into ℝ.

Note that in (1)–(3) above, *Q* compact implies that the constants *α*_0_, *λ*_0_, and *μ*_0_ may all be chosen independent of ***q*** ∈ *Q*. Furthermore, properties (1)–(3) immediately yield that the form *a*(***q***, ·, ·) defines a bounded, elliptic (i.e., *λ*_0_ = 0) operator A(q)∈L(V,V*) given by $\langle A(q)\hat{\varphi },\hat{\psi }\rangle =\langle A(q)(\varphi (0),\varphi ),(\psi (0),\psi )\rangle =-a(q,\varphi ,\psi )$ for $\hat{\varphi }=(\varphi (0),\varphi )$, $\hat{\psi }=(\psi (0),\psi )\in V$. If we define the set $D=\left\{\hat{\varphi }\in V:A(q)\hat{\varphi }\in H_{q}\right\}=\left\{(\varphi (0),\varphi )\in V:\varphi \in H^{2}(0,1),\varphi^{\prime }(1)=0\right\}$ which is independent of ***q*** for ***q*** ∈ *Q*, we obtain the closed, densely defined linear operator $A(q):D\subset H_{q}\to H_{q}$ given by $A(q)\hat{\varphi }=A(q)(\varphi (0),\varphi )=\left(q_{3}\varphi^{\prime }(0)-q_{4}\varphi (0),q_{1}\varphi^{\prime \prime }\right)$, $\hat{\varphi }=(\varphi (0),\varphi )\in D$. The operator $A(q):D\subset H_{q}\to H_{q}$ is regularly dissipative and (see, for example, [30]) is the infinitesimal generator of a holomorphic semigroup of bounded linear operators $\left\{e^{A(q)t}:t\geq 0\right\}$ on *H*_***q***_ and V*. Moreover, the system eq. (2.3) then has a state space form where $\frac{d\hat{v}}{dt}(t)=A(q)\hat{v}(t)+\left(\frac{q_{3}q_{2}}{q_{1}},0\right)u_{k}$ and $\hat{v}(k\tau )=\left(w_{k},x_{k}-\xi u_{k}\right)\;\text{for}\;k\tau <t<(k+1)\tau$. Then for time step *τ* > 0 and *k* = 0, 1, . . . , *K*, letting ${\hat{x}}_{k}=\left(w_{k},x_{k}\right)$, $\hat{A}(q)=e^{A(q)\tau }\in L\left(H_{q},H_{q}\right)$, and $\hat{B}(q)=(I-\hat{A}(q))\left\{(0,\xi )-A{\left(q\right)}^{-1}\left(\frac{q_{3}q_{2}}{q_{1}},0\right)\right\}\in L\left(\mathbb{R},H_{q}\right)$, it follows that

(2.5) $${\hat{x}}_{k+1}=\left(w_{k+1},x_{k+1}\right)=(w((k+1)\tau ),x((k+1)\tau ,\cdot ))=\hat{v}((k+1)\tau )+(0,\xi )u_{k}\;\;\;\;\;\;\;\;\;\;\;=e^{A(q)\tau }\left(w_{k},x_{k}-\xi u_{k}\right)+\int_{0}^{\tau }e^{A(q)s}\left(\frac{q_{3}q_{2}}{q_{1}},0\right)dsu_{k}+(0,\xi )u_{k}\;\;\;\;\;\;\;\;\;\;\;=\hat{A}(q){\hat{x}}_{k}+(I-\hat{A}(q))(0,\xi )u_{k}+A{\left(q\right)}^{-1}(\hat{A}(q)-I)\left(\frac{q_{3}q_{2}}{q_{1}},0\right)u_{k}\;\;\;\;\;\;\;\;\;\;=\hat{A}(q){\hat{x}}_{k}+\hat{B}(q)u_{k}$$

where for $\hat{B}(q)$ we have used the fact that the operator *A*(***q***)^−1^ commutes with the semigroup generated by *A*(***q***). Note that the operator $\hat{B}(q)$ is in fact an element in *H*_***q***_ and that (0, *ξ*) ∈ *V*, but that $\left(\frac{q_{3}q_{2}}{q_{1}},\;0\right)$ is only an element in *H*_***q***_. From eq. (2.5) the state space form of our discrete time model is

(2.6) $${\hat{x}}_{k+1}=\hat{A}(q){\hat{x}}_{k}+\hat{B}(q)u_{k},\;k=0,1,2,\ldots ,\;\;\;\;\;\;\;\;{\hat{x}}_{0}=\left(w_{0},\varphi_{0}\right),\;\;\;\;\;\;\;y_{k}=\hat{C}{\hat{x}}_{k},\;k=0,1,2,\ldots ,$$

where ${\hat{x}}_{k}=\left(w_{k},x_{k}\right)$, *k* = 0, 1, 2, . . . and the operator $\hat{C}\in L\left(H_{q},\mathbb{R}\right)$ is given by $\hat{C}(\theta ,\psi )=\theta$, where (*θ*, *ψ*) ∈ *H*_***q***_. Note that the ellipticity of A(q) guarantees the existence of *A*(***q***)^−1^.

From eq. (2.6) it is immediately clear that if we assume zero initial conditions, $\hat{x}=\left(w_{0},\varphi_{0}\right)=(0,0)$, the output *y* can be written as a discrete time convolution of the input, *u*, with a filter, *h*(***q***), as

(2.7) $$y_{k}=\sum_{j=0}^{k-1}\hat{C}\hat{A}{\left(q\right)}^{k-j-1}\hat{B}(q)u_{j}=\sum_{j=0}^{k-1}h_{k-j-1}(q)u_{j},\;k=0,1,2,\ldots ,$$

where for ***q*** ∈ *Q*, $h_{i}(q)=\hat{C}\hat{A}{\left(q\right)}^{i}\hat{B}(q)$, *i* = 0, 1, 2, . . .. Using the Trotter-Kato semigroup approximation theorem (see, for example, [31]), the following result can be shown (see, for proof, [32]).

#### Lemma 2.1.

*For Q a compact subset of the positive orthant or* ${\mathbb{R}}^{4}$, *K* = *Kτ for constant time step τ* > 0, *and h*_*i*_ *as defined in* eq. (2.7), *we have that the mapping* ***q*** ↦ *h*_*i*_(***q***) *from Q into* ℝ *is continuous, uniformly in* ***q*** *and i, for* ***q*** ∈ *Q and i* ∈ {0, 1, 2, . . . , *K*}.

Now although the input/output model given in eq. (2.7) is a standard convolution in ℝ, the filter, {*h*_*k*_(*q*)} involves the semigroup {*e*^*A*(*q*)*t*^ : *t* ≥ 0} which is defined on the infinite dimensional Hilbert space *H*_***q***_. Consequently, finite dimensional approximation is required. For *n* = 1, 2, . . . let ${\left\{\varphi_{i}^{n}(\eta )\right\}}_{i=0}^{n}$ denote the standard linear B-splines on the interval [0, 1] defined with respect to the uniform mesh $\left\{0,\frac{1}{n},\frac{2}{n},\ldots ,\frac{n-1}{n},1\right\}$, $\varphi_{i}^{n}(\eta )=(n\eta -i+1)1_{\left[\frac{i-1}{n},\frac{i}{n}\right]}+(1-n\eta +i)1_{\left[\frac{i}{n},\frac{i+1}{n}\right]}$. Set

(2.8) $$V^{n}=\mathrm{span}\left\{{\hat{\varphi }}_{i}^{n}\right\}=\mathrm{span}{\left\{\left(\varphi_{i}^{n}(0),\varphi_{i}^{n}\right)\right\}}_{i}^{n}$$

and let $P_{q}^{n}:H_{q}\to V^{n}$ denote the orthogonal projection of *H*_***q***_ on to *V*^*n*^ along (*V*^*n*^)^⊥^. Standard arguments from the theory of splines (see, for example, [33]) can be used to argue that $|P_{q}^{n}(\theta ,\psi )-{\left(\theta ,\psi \right)|}_{q}\to 0$, as *n* → ∞, for all (*θ*, *ψ*) ∈ *H*_***q***_, and that ${\left\|P_{q}^{n}\hat{\varphi }-\hat{\varphi }\right\|}_{V}\to 0$, as *n* → ∞, for all $\hat{\varphi }\in V$ with the convergence uniform in ***q*** for ***q*** ∈ *Q*.

For *n* = 1, 2, ... and *k* = 0, 1, 2, ... we set ${\hat{x}}_{k}^{n}(\eta )=\sum_{i=0}^{n}X_{i}^{n,k}{\hat{\varphi }}_{i}^{n}(\eta )$, and we approximate the operator *A*(***q***) using a Galerkin approach. That is, we define the operator $A^{n}(q)\in L\left(V^{n},V^{n}\right)$ by restricting the form *a*(***q***, ·, ·) to *V*^*n*^ × *V*^*n*^. We then set

(2.9) $${\hat{A}}^{n}(q)=e^{A^{n}(q)\tau },\;\;\text{and}\;{\hat{B}}^{n}(q)=\left(I^{n}-{\hat{A}}^{n}(q)\right)\left\{(0,\xi )-A^{n}{\left(q\right)}^{-1}P_{q}^{n}\left(\frac{q_{3}q_{2}}{q_{1}},0\right)\right\},$$

where ${\hat{B}}^{n}(q)\in L\left(\mathbb{R},V^{n}\right)=V^{n}$. The matrix representations for these operators with respect to the basis ${\left\{{\hat{\varphi }}_{i}^{n}\right\}}_{i=0}^{n}$ are then given by $\left[A^{n}(q)\right]=-{\left[M^{n}(q)\right]}^{-1}K^{n}(q)$, $\left[{\hat{A}}^{n}(q)\right]=e^{-{\left[M^{n}(q)\right]}^{-1}K^{n}(q)\tau }$, and $\left[{\hat{B}}^{n}(q)\right]=\left(I-\left[{\hat{A}}^{n}(q)\right]\right)\left\{\Xi^{n}-{\left[A^{n}(q)\right]}^{-1}{\left[M^{n}(q)\right]}^{-1}{\left[q_{2},0,0,\ldots ,0\right]}^{T}\right\}=\left(I-\left[{\hat{A}}^{n}(q)\right]\right)\left\{\Xi^{n}+{\left[K^{n}(q)\right]}^{-1}{\left[q_{2},0,0,\ldots ,0\right]}^{T}\right\}$, where ${\left[M\right]}_{i,j=0}^{n}={\langle \left(\varphi_{i}^{n}(0),\varphi_{i}^{n}\right),\left(\varphi_{j}^{n}(0),\varphi_{j}^{n}\right)\rangle }_{q}$, ${\left[K\right]}_{i,j=0}^{n}=a\left(q,\varphi_{i}^{n}(0),\varphi_{i}^{n}\right)$, $\left(\varphi_{j}^{n}(0),\varphi_{j}^{n}\right))$, and $\Xi^{n}=\frac{q_{2}}{q_{1}}{\left[0,\frac{1}{n},\frac{2}{n},\ldots ,\frac{n-1}{n},1\right]}^{T}$. Letting $\left[{\hat{C}}^{n}\right]=[1,0,0,\ldots ,0]\in {\mathbb{R}}^{1\times (n+1)}$, we consider the discrete time dynamical system in *V*^*n*^ given by

(2.10) $${\hat{x}}_{k+1}^{n}={\hat{A}}^{n}(q){\hat{x}}_{k}^{n}+{\hat{B}}^{n}(q)u_{k},\;k=0,1,2,\ldots ,K-1\;\;\;\;\;\;\;y_{k}^{n}=\hat{C}{\hat{x}}_{k}^{n},\;k=0,1,2,\ldots ,K,\;\;\;\;\;\;{\hat{x}}_{0}^{n}=(0,0)\in V^{n}$$

or equivalently in ${\mathbb{R}}^{n+1}$ given by the system where $X^{n,k+1}=\left[{\hat{A}}^{n}(q)\right]X^{n,k}+\left[{\hat{B}}^{n}(q)\right]u_{k}$, $y_{k}^{n}=\left[{\hat{C}}^{n}\right]X^{n,k}$, and $X^{n,0}={\left[0,0,\ldots ,0\right]}^{T}\in {\mathbb{R}}^{n+1}$, we obtain that

(2.11) $$\begin{array}{l} y_{k}^{n}=\sum_{j=0}^{k-1}{\hat{C}}^{n}{\hat{A}}^{n}{\left(q\right)}^{k-j-1}{\hat{B}}^{n}(q)u_{j}\\ \;\;\;\;\;\;\;=\sum_{j=0}^{k-1}\left[{\hat{C}}^{n}\right]{\left[{\hat{A}}^{n}(q)\right]}^{k-j-1}\left[{\hat{B}}^{n}(q)\right]u_{j}\\ \;\;\;\;\;\;\;=\sum_{j=0}^{k-1}h_{k-j-1}^{n}(q)u_{j}, \end{array}$$

for *k* = 0, 1, 2, . . . , *K* where $h_{i}^{n}(q)=\left[{\hat{C}}^{n}\right]{\left[{\hat{A}}^{n}(q)\right]}^{i}\left[{\hat{B}}^{n}(q)\right]\in \mathbb{R}$, *i* = 0, 1, 2, . . . , *K* − 1.

Using linear semigroup theory (see, for example, [21, 34, 35]) and in particular the Trotter-Kato semigroup approximation theorem (see, for example, [36] and [31]) the following results can be established (for proof, see [32]).

#### Theorem 2.1.

*For Q a compact subset of the positive orthant of ${\mathbb{R}}^{4}$, $n=1,2,\ldots ,{\left\{\varphi_{i}^{n}(\eta )\right\}}_{i=0}^{n}$ the standard linear B-splines on the interval* [0, 1] *defined with respect to the uniform mesh $\left\{0,\frac{1}{n},\frac{2}{n},\ldots ,\frac{n-1}{n},1\right\}$, $V^{n}=span{\left\{\left(\varphi_{i}^{n}(0),\varphi_{i}^{n}\right)\right\}}_{i}^{n}$, $P_{q}^{n}:H_{q}\to V^{n}$ the orthogonal projection of H*_***q***_ *on to V*^*n*^ *along* (*V*^*n*^)^⊥^, *and ${\hat{A}}^{n}(q)$ and ${\hat{B}}^{n}(q)$ defined as in* eq. (2.9), *we have that* ${\left|{\hat{A}}^{n}(q)P_{q}^{n}(\theta ,\psi )-\hat{A}(q)(\theta ,\psi )\right|}_{q}\to 0$, *as n* → ∞, *for all* (*θ*, *ψ*) ∈ *H*_***q***_, *that* ${\left\|{\hat{A}}^{n}(q)P_{q}^{n}\hat{\varphi }-\hat{A}(q)\hat{\varphi }\right\|}_{V}\to 0$, *as n* → ∞, *for all* $\hat{\varphi }\in V$, *and that* ${\left\|{\hat{B}}^{n}(q)-\hat{B}(q)\right\|}_{V}\to 0$, *as n* → ∞, *with the convergence in all cases uniform in* ***q*** *for* ***q*** ∈ *Q*.

#### Theorem 2.2.

*Under the same hypotheses as Theorem 2.1, we have that ${\left\|{\hat{x}}_{k}^{n}(q)-{\hat{x}}_{k}(q)\right\|}_{V}\to 0$, that ${\left|{\hat{x}}_{k}^{n}(q)-{\hat{x}}_{k}(q)\right|}_{q}\to 0$, that $\left|y_{k}^{n}-y_{k}\right|\to 0$, and that $\left|h_{k}^{n}(q)-h_{k}(q)\right|\to 0$ as n* → ∞ *uniformly in k for k* ∈ {0, 1, 2, . . . , *K*} *and uniformly in* ***q*** *for* ***q*** ∈ *Q.*

Finally, we will assume that we have training data, ${\left\{{\left\{u_{k}^{i}\right\}}_{k=0}^{K},\;{\left\{y_{k}^{i}\right\}}_{k=0}^{K}\right\}}_{i=1}^{R}$, from *R* participants or subjects where without loss of generality (i.e., by padding with zeros) we have assumed that all training input/output datasets have the same number, *K*, of observations. In this case, for *i* = 1 , . . . , *R* we have,

(2.12) $$y_{k}^{i}=\sum_{j=0}^{k-1}h_{k-j-1}(q)u_{j}^{i},\;\;\text{and}\;y_{k}^{n,i}=\sum_{j=0}^{k-1}h_{k-j-1}^{n}(q)u_{j}^{i},\;k=0,1,\ldots ,K,$$

where $h_{j}(q)=\hat{C}\hat{A}{\left(q\right)}^{j}\hat{B}(q)\in \mathbb{R}$ and $h_{j}^{n}(q)=\left[{\hat{C}}^{n}\right]{\left[{\hat{A}}^{n}(q)\right]}^{j}\left[{\hat{B}}^{n}(q)\right]\in \mathbb{R}$, for *j* = 0, 1, . . . , *K* − 1. This formulation facilitates the estimation of the population parameters ***q***. If one wishes to find the parameters ***q*** for a specific individual, the methods outlined in Section 2.2 can still be applied by letting the indices *i* = 1, . . . , *R* refer to different measured BrAC/TAC events each with *k* = 0, . . . , *K* denoting the measurement times for the desired individual subject.

### 2.2. Bayesian estimation of dynamical system parameters

In this section we develop a Bayesian framework to estimate the unknown parameters ***q*** = [*q*_1_, *q*_2_]^*T*^ in the system eq. (2.2). To illustrate our approach, for simplicity but without loss of generality, we have assumed that the sensor parameters *q*_3_ and *q*_4_ have been bench-measured and are therefore known and concentrate our effort on estimating the physiological subject-dependent parameters *q*_1_ and *q*_2_. All of what follows below can easily be extended to estimating all four of the parameters in the model. Our underlying statistical model incorporating noise is based on the observation of $\left\{y_{j}^{i}\right\}$ as in eq. (2.12) and is given by

(2.13) $$V_{j}^{i}=y_{j}^{i}+\varepsilon_{j}^{i}=\sum_{\ell =0}^{j-1}h_{j-\ell -1}u_{\ell }^{i}+\varepsilon_{j}^{i}$$

where $V_{j}^{i}$ are our measured TAC values, and $\varepsilon_{j}^{i}$ are the i.i.d. noise terms corresponding to person *i* at time *jτ* with *σ* > 0, *τ* > 0. Commonly, as we will assume in Section 3.2 and beyond, $\varepsilon_{j}^{i}\sim N\left(0,\sigma^{2}\right)$. In order to be able to carry out the requisite computations, we consider the approximating statistical model based on eq. (2.12)

(2.14) $$V_{j}^{i}=y_{j}^{n,i}+\varepsilon_{j}^{i}=\sum_{\ell =0}^{j-1}h_{j-\ell -1}^{n}u_{\ell }^{i}+\varepsilon_{j}^{i},$$

where once again the Vji's are assumed to be the measured TAC values. We consider q to be a random vector on some probability space {Ω, Σ, *P*} with support *Q* and assume that the prior distribution of q is given by the push forward measure *π*_0_. That is for *A* ⊂ *Q*, $P(\{q\in A\})=P\left(q^{-1}(A)\right)=\int_{q^{-1}(A)}dP=\int_{A}d\pi_{0}(q)$.

We assume independence across both *i* (individuals) and *j* (sampling times for each individual), for each *i* and *j* we have $V_{j}^{i}-y_{j}^{i}=\varepsilon_{j}^{i}$ (commonly distributed *N*(0, *σ*^2^)) and similarly, $V_{j}^{i}-y_{j}^{n,i}=\varepsilon_{j}^{i}$ (again commonly distributed *N*(0, *σ*^2^)). Letting *φ* denote the density of εji's, for ***q*** ∈ *Q* the likelihood and the approximating likelihood functions are given respectively by (see, for example, [37–40])

$$L\left(q|\left\{V_{j}^{i}\right\}\right)=\prod_{i=1}^{R}\prod_{j=1}^{K}\varphi \left(V_{j}^{i}-y_{j}^{i}\right),\;\;\text{and}$$

$$L^{n}\left(q|\left\{V_{j}^{i}\right\}\right)=\prod_{i=1}^{R}\prod_{j=1}^{K}\varphi \left(V_{j}^{i}-y_{j}^{n,i}\right).$$

An application of Bayes’ Theorem (see, for example, Theorem 1.31 in [41]) yields that the posterior distribution of q or the conditional distribution of q conditioned on the data, $\left\{V_{j}^{i}\right\}$, is a push forward measure $\pi =\pi \left(\cdot |\left\{V_{j}^{i}\right\}\right)$ that is absolutely continuous with respect to *π*_0_ and whose Radon-Nikodym derivative, or density, for ***q*** ∈ *Q* is given by

(2.15) $$\frac{d\pi }{d\pi_{0}}\left(q|\left\{V_{j}^{i}\right\}\right)=\frac{L\left(q|\left\{V_{j}^{i}\right\}\right)}{\int_{Q}L\left(q|\left\{V_{j}^{i}\right\}\right)d\pi_{0}(q)}=\frac{1}{Z}\prod_{i=1}^{R}\prod_{j=1}^{K}\varphi \left(V_{j}^{i}-y_{j}^{i}(q)\right),\;\text{where}\;$$

(2.16) $$Z=\int_{Q}L\left(q|\left\{V_{j}^{i}\right\}\right)d\pi_{0}(q)=\int_{Q}\prod_{i=1}^{R}\prod_{j=1}^{K}\varphi \left(V_{j}^{i}-y_{j}^{i}(q)\right)d\pi_{0}(q).$$

In this way, for *A* ⊂ *Q*, we have $P\left(q\in A|\left\{V_{j}^{i}\right\}\right)=\int_{A}d\pi (q)=\int_{A}d\pi \left(q|\left\{V_{j}^{i}\right\}\right)$. If, in addition, we have *π*_0_ ≪ *λ* with density $\frac{d\pi_{0}}{d\lambda }=f_{0}$ where *λ* denotes Lebesgue measure on *Q*, then *π* ≪ *λ* with conditional density *f* given by

(2.17) $$f(q)=\frac{d\pi }{d\lambda }\left(q|\left\{V_{j}^{i}\right\}\right)=\frac{L\left(q|\left\{V_{j}^{i}\right\}\right)f_{0}(q)}{\int_{Q}L\left(q|\left\{V_{j}^{i}\right\}\right)f_{0}(q)d\lambda (q)}=\frac{1}{Z}\prod_{i=1}^{R}\prod_{j=1}^{K}\varphi \left(V_{j}^{i}-y_{j}^{i}(q)\right)f_{0}(q),$$

(2.18) $$Z=\int_{Q}L\left(q|\left\{V_{j}^{i}\right\}\right)f_{0}(q)d\lambda (q)=\int_{Q}\prod_{i=1}^{R}\prod_{j=1}^{K}\varphi \left(V_{j}^{i}-y_{j}^{i}(q)\right)f_{0}(q)d\lambda (q),\;\;\text{and}\;$$

(2.19) $$P\left(\{q\in A\}|\left\{V_{j}^{i}\right\}\right)=\int_{A}f(q)d\lambda (q)=\int_{A}f\left(q|\left\{V_{j}^{i}\right\}\right)d\lambda (q).$$

Analogously, in the case of the approximating likelihood eq. (2.15), eq. (2.16), eq. (2.17), eq. (2.18), and eq. (2.19) respectively become

(2.20) $$\frac{d\pi^{n}}{d\pi_{0}}\left(q|\left\{V_{j}^{i}\right\}\right)=\frac{L^{n}\left(q|\left\{V_{j}^{i}\right\}\right)}{\int_{Q}L^{n}\left(q|\left\{V_{j}^{i}\right\}\right)d\pi_{0}(q)}=\frac{1}{Z}\prod_{i=1}^{R}\prod_{j=1}^{K}\varphi \left(V_{j}^{i}-y_{j}^{n,i}(q)\right),$$

(2.21) $$Z^{n}=\int_{Q}L^{n}\left(q|\left\{V_{j}^{i}\right\}\right)d\pi_{0}(q)=\int_{Q}\prod_{i=1}^{R}\prod_{j=1}^{K}\varphi \left(V_{j}^{i}-y_{j}^{n,i}(q)\right)d\pi_{0}(q),$$

(2.22) $$f^{n}(q)=\frac{L^{n}\left(q|\left\{V_{j}^{i}\right\}\right)f_{0}(q)}{\int_{Q}L^{n}\left(q|\left\{V_{j}^{i}\right\}\right)f_{0}(q)d\lambda (q)}=\frac{1}{Z^{n}}\prod_{i=1}^{R}\prod_{j=1}^{K}\varphi \left(V_{j}^{i}-y_{j}^{n,i}(q)\right)f_{0}(q),$$

$Z^{n}=\int_{Q}L^{n}\left(q|\left\{V_{j}^{i}\right\}\right)f_{0}(q)d\lambda (q)=\int_{Q}\prod_{i=1}^{K}\prod_{j=1}^{K}\varphi \left(V_{j}^{i}-y_{j}^{n,i}(q)\right)f_{0}(q)d\lambda (q)$ and $P\left(q\in A|\left\{V_{j}^{i}\right\}\right)=\int_{A}f^{n}(q)d\lambda (q)=\int_{A}f^{n}\left(q|\left\{V_{j}^{i}\right\}\right)d\lambda (q)$

## 3. Results

### 3.1. Convergence in distribution

Consider the random variable q with posterior distribution described by the measure *π* given in eq. (2.15) and eq. (2.16), and let $q^{n}$ denote the random variable q but with posterior distribution *π*^*n*^ given by eq. (2.20) and eq. (2.21). In this section we establish that $q^{n}\overset{\;\text{Dist}\;}{\to }q$ as *n* → ∞; that is that $q^{n}$ converges in distribution to q. Recall that due to the physical constraints based on our model for the alcohol biosensor problem, eq. (2.2), we require that the parameters ***q*** lie in the interior of the positive orthant of ${\mathbb{R}}^{2}$.

#### Theorem 3.1.

*For Q a compact set in the interior of the positive orthant of ${\mathbb{R}}^{2}$, a prior π*_0_ *with compact support Q and a density that is continuous on Q, and a noise distribution with bounded density function φ and support on ℝ, $q^{n}$, the random variable with posterior distribution π*^*n*^ *given by* eq. (2.20) *and* eq. (2.21) *converges in distribution to the random variable* q *with posterior distribution π given by* eq. (2.15) *and* eq. (2.16).

##### Proof.

For *S* a subset *Q*, the triangle inequality yields

(3.1) $$\left|P\left(q^{n}\in S|\left\{V_{j}^{i}\right\}\right)-P\left(q\in S|\left\{V_{j}^{i}\right\}\right)\right|=\left|\frac{1}{Z^{n}}\int_{S}L^{n}\left(q|\left\{V_{j}^{i}\right\}\right)d\pi_{0}(q)-\frac{1}{Z}\int_{S}L\left(q|\left\{V_{j}^{i}\right\}\right)d\pi_{0}(q)\right|\;\;\;\;\;\;\;\;\;\;\;\;\;\;\leq \left|\frac{1}{Z}-\frac{1}{Z^{n}}\right|\left(\int_{S}L\left(q|\left\{V_{j}^{i}\right\}\right)d\pi_{0}(q)\right)+\left(\frac{1}{Z^{n}}\right)\int_{S}\left|L\left(q|\left\{V_{j}^{i}\right\}\right)-L^{n}\left(q|\left\{V_{j}^{i}\right\}\right)\right|d\pi_{0}(q),$$

where *φ* is the normal density describing the distribution of the noise term in eq. (2.13), and *Z* and *Z*^*n*^ are as in eq. (2.16) and eq. (2.21), respectively.

Focusing first on the limit of |1/*Z* − 1/*Z*^*n*^| as *n* → ∞, by Lemma 2.1 we have that the $y_{i}^{j}(q)$ are continuous in ***q*** for ***q*** ∈ *Q*, *i* ∈ {0, 1, . . . , *R*}, and *j* ∈ {0, 1, . . . , *K*}. Since *Q* is compact, the $\left\{y_{i}^{j}(q)\right\}$ are bounded and thus 0 < *Z* < ∞. By Theorem 2.2, since $y_{j}^{n,i}(q)\to y_{j}^{i}(q)$ uniformly in ***q*** for ***q*** ∈ *Q* as *n* → ∞, 0 < *Z*^*n*^ < ∞ for *n* large enough. Again by Theorem 2.2 it follows from eq. (2.16), eq. (2.21), and the Bounded Convergence Theorem that *Z*^*n*^ → *Z* as *n* → ∞ and therefore that |1/*Z* − 1/*Z*^*n*^| → 0 as *n* → ∞. Then, essentially the same arguments yield that $\int_{S}\left|L\left(q|\left\{V_{j}^{i}\right\}\right)-L^{n}\left(q|\left\{V_{j}^{i}\right\}\right)\right|d\pi_{0}(q)\to 0$, from which it then immediately follows that $\left(\frac{1}{Z^{n}}\right)\int_{S}\left|L\left(q|\left\{V_{j}^{i}\right\}\right)-L^{n}\left(q|\left\{V_{j}^{i}\right\}\right)\right|d\pi_{0}(q)\to 0$, and therefore from eq. (3.1) that $q^{n}\overset{\;\text{Dist}\;}{\to }q$ as *n* → ∞ and the theorem has been proved. □

For the push forward measures *π* and *π*^*n*^ from eq. (2.15) and eq. (2.20), respectively, we are commonly interested in their respective expected values and the convergences there within. Since *Q* is compact, a direct invocation of the Portmanteau theorem (see, [41]) establishes the following corollary which guarantees the convergence of all moments described by *π*^*n*^ to those of *π*.

#### Corollary 3.1.

*Under the hypotheses of* Theorem 3.1, *and for any continuous function* g:Q→ℝ *we have that* $E_{\pi^{n}}[g(q)]=\int_{Q}g(q)d\pi^{n}(q)\to \int_{Q}g(q)d\pi (q)=E_{\pi }[g(q)]$ *as n* → ∞.

### 3.2. Consistency

In this section we demonstrate the strong consistency of the posterior distribution with respect to the parameters, ***q***, by imposing stronger assumptions on the distribution of the noise terms $\varepsilon_{j}^{i}$ in eq. (2.13) and on the prior, *π*_0_, by restricting *Q* to a rectangle in the positive orthant of ${\mathbb{R}}^{2}$, and by applying the framework summarized in [42].

As in [42], we show consistency of the posterior distribution *π* as in eq. (2.17), rather than consistency of a point estimator based on the posterior distribution. As such, for prior *π*_0_ over *Q*, posterior $\pi \left(\cdot |\left\{V_{j}^{i}\right\}\right)$ as in eq. (2.15), and i.i.d. noise $\varepsilon_{j}^{i}\sim N\left(0,\sigma^{2}\right)$ for *σ* > 0, we also consider that for ***q*** ∈ *Q* assumed known we have random variables $V_{j}^{i}\sim N\left(y_{j}^{i}(q),\sigma^{2}\right)$ as determined by eq. (2.13) for *i* = 1, 2, . . . , *R* and *j* = 1, 2, . . . , *K*. Further, we have that ${\left\{V_{j}^{i}\right\}}_{i,j}$ are independent in *i* and *j*, but are non-identically distributed (i.n.i.d).

For clarity and brevity we consider the random vector $V^{i}={\left(V_{0}^{i},V_{1}^{i},\ldots ,V_{K}^{i}\right)}^{T}$ with values in ${\mathbb{R}}^{K+1}$ and independent entries derived from the matrix equivalent to eq. (2.13) and eq. (2.14), namely ***V***^*i*^ = ***y***^*i*^ + ***ε***^*i*^ = ***Hu***^*i*^ + ***ε***^*i*^ and ***V***^*i*^ = ***y***^*n*,*i*^ + ***ε***^*i*^ = ***H***^*n*^***u***^*i*^ + ***ε***^*i*^, with noise vectors $\varepsilon^{i}={\left(\varepsilon_{0}^{i},\varepsilon_{1}^{i},\ldots ,\varepsilon_{K}^{i}\right)}^{T}$, observed TAC vectors $V^{i}={\left(V_{0}^{i},V_{1}^{i},\ldots ,V_{K}^{i}\right)}^{T}$, theoretical TAC vectors $y^{i}={\left(y_{0}^{i},j_{1}^{i},\ldots ,y_{K}^{i}\right)}^{T}$ and analogous ***y***^*n*,*i*^, BrAC data vectors $u^{i}={\left(u_{0}^{i},u_{1}^{i},\ldots ,u_{K}^{i}\right)}^{T}$ and analogous ***u***^*n*,*i*^, and kernel matrices *H* and *H*^*n*^ with entries ${\left[H\right]}_{i,j}=h_{i-j}1_{j\leq i}$ and ${\left[H^{n}\right]}_{i,j}=h_{i-j}^{n}1_{j\leq i}$, respectively. In this way, for every *i* ∈ {1, 2, . . . , *R*}, by independence in *j* we have the family of joint distributions, $\left\{f_{i,q}(\cdot )=\prod_{j=0}^{K}\varphi \left(\cdot_{j}-y_{j}^{i}(q)\right):q\in Q\right\}$, representing the possible densities of $V^{i}$ for *φ* the noise density and $y_{j}^{i}$ as in eq. (2.12). We are interested in the scenario where the number of subjects *R* → ∞.

With our reframing, for *A* ⊂ *Q* by independence in *i* we may rewrite eq. (2.15) as

(3.2) $$\pi \left(A|\left\{V^{i}\right\}\right)=\frac{\int_{A}\prod_{i=1}^{R}f_{i,q}\left(V^{i}\right)d\pi_{0}(q)}{\int_{Q}\prod_{i=1}^{R}f_{i,q}\left(V^{i}\right)d\pi_{0}(q)}=\frac{\int_{A}L\left(q|\left\{V^{i}\right\}\right)d\pi_{0}(q)}{\int_{Q}L\left(q|\left\{V^{i}\right\}\right)d\pi_{0}(q)}=\frac{\int_{A}\frac{L\left(q|\left\{V^{i}\right\}\right)}{L\left(q_{0}|\left\{V^{i}\right\}\right)}d\pi_{0}(q)}{\int_{Q}\frac{L\left(q|\left\{V^{i}\right\}\right)}{L\left(q_{0}|\left\{V^{i}\right\}\right)}d\pi_{0}(q)}=\frac{J_{A}\left(\left\{V^{i}\right\}\right)}{J\left(\left\{V^{i}\right\}\right)},$$

where for all *i* we have data vectors ***V***^*i*^, and for our purposes we will be interested in the equivalent form on the right-hand side of the equation above where ***q***_0_ ∈ *Q* is the true value of our parameters [*q*_1_, *q*_2_]^*T*^.

We first formalize the results discussed in Section 7 of [42] that handle the i.n.i.d case of posterior consistency. As such, we say that our posterior distributions {*π*(·|{***V***^*i*^})} as in eq. (3.2) are *strongly consistent at* ***q***_0_ if {*π*(*U*|{***V***^*i*^})} → 1 a.s $P_{q_{0}}^{\infty }$ for every neighborhood *U* of ***q***_0_ as *R* → ∞, where $P_{q_{0}}^{\infty }=\prod_{i=1}^{\infty }P_{i,q_{0}}$ with $P_{i,q_{0}}$ the probability distribution generated by $f_{i,q_{0}}$ with data samples {***V***^*i*^}.

For this we show that for sets *A* ⊂ *Q* with ***q***_0_ ∉ *A*, *J*_*A*_({*V*_*k*_}) → 0 and *J*({***V***^*i*^}) → ∞ as *R* → ∞ in some appropriate manner to be made precise below. For *J*_*A*_({***V***^*i*^}) → 0 we take the same approach as expressed in [42] and thus state the following definition below without motivation, where we note that for any two densities *f*, *g* on some space X their ***affinity***, denoted Aff(*f*, *g*), is given by $\mathrm{Aff}(f,g)=\int_{X}\sqrt{f(x)g(x)}dx$.

#### Definition 3.1.

Let *A* ⊂ *Q* and *δ* > 0. The set *A* and ***q***_0_ are **strongly** *δ* **separated** if for every probability measure *ν* on *A*, $\mathrm{Aff}\left(f_{1,q_{0}},v_{v}^{1}\right)<\delta$ where $f_{1,q_{0}}(x)=\prod_{j=0}^{K}\varphi \left(x_{j}-y_{j}^{1}\left(q_{0}\right)\right)$ for $x\in {\mathbb{R}}^{K+1}$ as in the work surrounding eq. (3.2), and $v_{v}^{R}$ is the marginal density of ${\left\{V^{i}\right\}}_{i=1}^{R}$ given by $v_{v}^{R}\left({\left\{V^{i}\right\}}_{i=1}^{R}\right)=\int_{A}L\left(q|{\left\{V^{i}\right\}}_{i=1}^{R}\right)dv(q)$ for any *R* = 1, 2, . . .. We will say that *A* and ***q***_0_ are **strongly separated** if they are strongly *δ* separated for some *δ* > 0.

From these definitions we provide the following theorem without proof. For proof see Sections 3 and 7 of [42] (or, for examples, [43])

#### Theorem 3.2.

*Let π*_0_ *be a prior over parameter space Q*, ${\left\{V^{i}\right\}}_{i=1}^{\infty }$ *be independent but not identically distributed data with distribution generated by f*_i,**q**_ *for* ***q*** ∈ *Q,* ***q***_0_ ∈ *Q the true value of the parameters* [*q*_1_, *q*_2_]^*T*^, *and A* ⊂ *Q with* ***q***_0_ ∉ *A. If* $A=\underset{i\geq 1}{\cup }A_{i}$ *such that*

1. *For some δ* > 0, *all A*_*i*_*’s are strongly δ separated from* ***q***_0_ *for the model* ***q*** ⟼ *f*_i,**q**_, *and*
2. $\sum_{i\geq 1}\sqrt{\pi_{0}\left(A_{i}\right)}<\infty$,

*then for some β*_0_ > 0, $e^{R\beta_{0}}J_{A}\left({\left\{V^{i}\right\}}_{i=1}^{R}\right)\to 0$ *a.s*. $P_{q_{0}}^{\infty }$ *as R* → ∞ *for J*_*A*_({***V***^*i*^}) *as in* eq. (3.2).

To show *J*({***V***^*i*^}) → ∞ as *R* → ∞ we utilize the approach as outlined in the proof Theorem 1 of Appendix A.2 in [44] (specifically the proof of (8) in Appendix A.2). For a direct proof see [32]. For a similar approach see [45].

#### Theorem 3.3.

*Let π*_0_ *be a prior over parameter space Q*, ${\left\{V^{i}\right\}}_{i=1}^{\infty }$ *be independent but not identically distributed data with distribution generated by f*_i,**q**_ *for* ***q*** ∈ *Q, and q*_0_ ∈ *Q the true value of the parameters* [***q***_1_, ***q***_2_]^*T*^*. For* ***q*** ∈ *Q define* $\Lambda_{i}\left(q_{0},q\right)=\log\frac{f_{i,q_{0}}\left(V^{i}\right)}{f_{i,q}\left(V^{i}\right)}$, $K_{i}\left(q_{0},q\right)=E_{q_{0}}\left[\Lambda_{i}\left(q_{0},q\right)\right]$, *and* $S_{i}\left(q_{0},q\right)={\mathrm{Var}}_{q_{0}}\left[\Lambda_{i}\left(q_{0},q\right)\right]$. *If there exists a set B* ⊆ *Q with π*_0_(*B*) > 0 *such that*

1. $\sum_{i\geq 1}\frac{S_{i}\left(q_{0},q\right)}{i^{2}}<\infty$ **∀*q*** ∈ *B, and*
2. *For every ε* > 0, $\pi_{0}\left(B\cap \left\{q:K_{i}\left(q_{0},q\right)<\varepsilon \;\forall i\right\}\right)>0$.

*Then* $\forall \beta >0,e^{R\beta }J\left({\left\{V^{i}\right\}}_{i=1}^{R}\right)\to \infty$ *a.s*. $P_{q_{0}}^{\infty }$ *as R* → ∞ *for J*({***V***^*i*^}) *as in* eq. (3.2).

Before moving on to our main theorem we apply the following theorem and subsequent corollary to prove a lemma that will be of use to us later. For proof of the following see Theorem 5.3 of [46].

#### Theorem 3.4.

*For parameters* ***q*** ∈ *Q a subset of the positive orthant of* ${\mathbb{R}}^{n}$, *and* ***q****-dependent semigroup* {*T*(·; ***q***) : *t* > 0} *with infinitesimal generator A*(***q***) *defined in terms of a* ***q****-dependent sesquilinear form* σ(q):V×V→ℂ *on a Hilbert space V satisfying items 1 and 2 in Section 2.1, assume*

1. (***Affine****) The map* ***q*** ↦ *σ*(***q***) *is affine, in the sense that for any u*, *v* ∈ *V, σ*(***q***, *u*, *v*) = *σ*_0_(*u*, *v*) + *σ*_1_(***q***, *u*, *v*) *where σ*_0_ *is independent of* ***q*** *and the map* ***q*** ↦ *σ*_1_(***q***, ·, ·) *is linear, and*
2. (***Continuous****) For any* ***q***, $\bar{q}\in q$ *with metric d*_*Q*_(·, ·) *we have the bound $|\sigma (q,u,v)-\sigma (\bar{q},u,v)|\;\leq \;d_{q}(q,\bar{q})\|u{\|}_{V}\|v{\|}_{V}$, for all u*, *v* ∈ *V.*

*Then, the semigroup T*(·; ***q***) *is (Fréchet) di*ff*erentiable in* ***q*** *in the interior of Q, where for t* > 0, $\bar{q}\in q$, *and acting on δ****q*** ∈ *Q the derivative is given by*

(3.3) $$T_{q}(t,\bar{q})\delta q=\frac{1}{2\pi i}\int_{\partial \Sigma_{\gamma }}e^{\lambda t}R(\lambda ,q)A(\delta q)R(\lambda ,q)d\lambda$$

*for R*(*λ*, ***q***) = (*A*(***q***) − *λI*)^−1^ *the resolvent of A*(***q***), *and the obtuse sector* $\Sigma_{\gamma }=\{\lambda \in \mathbb{C}:\arg\left(\lambda -\lambda_{0}\right)\leq \frac{\pi }{2}+\arctan\left(\left(1+\alpha_{0}\mu_{0}\right)(1-\gamma )\right)\}$ *with* γ ∈ (0, 1), *α*_0_ *as in item 1, and λ*_0_, *μ*_0_ *as in item 2.*

The following corollary is an immediate consequence of the work in [6]. Specifically that the map ***q*** ↦ *R*(*λ*, ***q***) is analytic as a map from *Q* to L(V*,V), and from eq. (3.3) the map $\bar{q}\mapsto T_{q}(t,\bar{q})$ depends continuously on $R(\lambda ,\bar{q})$. Note that *Σ*_γ_ is independent of ***q*** as the constants *α*_0_ and *λ*_0_, *μ*_0_ from items 1 and 2, respectively, of Section 2.1 are independent of ***q***.

#### Corollary 3.2.

Under the same hypotheses as Theorem 3.4, we have that the map $\bar{q}\mapsto T_{q}(t,\bar{q})$ is continuous in the operator norm on L(q,L(V*,V)) for $\bar{q}$ in the interior of Q.

#### Lemma 3.1.

*For Q a rectangle in the positive orthant of* ${\mathbb{R}}^{2}$, *Hilbert spaces H*_*q*_ *and V as in* eq. (2.4), *bilinear form* a(q,⋅,⋅):V×V→ℝ *as in* Section 2.1 *with q*_3_ *and q*_4_ *assumed known, and induced infinitesimal generator A*(***q***) *as in* Section 2.1, *then the generated holomorphic semigroup of bounded linear operators* $\left\{e^{A(q)t}:t\geq 0\right\}$ *on H*_***q***_ and V* *is (Fréchet) differentiable and Lipschitz in* ***q*** *in the interior of Q. Further, for i* = 1, . . . , *R and j* = 1, . . . , *K*, $y_{j}^{i}$ *and* $y_{j}^{n,i}$ *as in* eq. (2.12) *and* eq. (2.12) *are (Fréchet) differentiable and Lipschitz in* ***q*** *with Lipschitz constants independent of i and j.*

##### Proof.

First, by item 3 of Section 2.1 we have that for ***q*** ∈ *Q*, ***q*** ↦ *a*(***q***, ·, ·) is continuous in ***q***. Second, notice that for $\hat{\psi }$, $\hat{\varphi }\in V$ with $\hat{\psi }=(\psi (0),\psi )$ and $\hat{\varphi }=(\varphi (0),\varphi )$, we have $a(q,\hat{\psi },\hat{\varphi })=\frac{q_{1}q_{4}}{q_{3}}\psi (0)\varphi (0)+q_{1}\int_{0}^{1}\psi^{\prime }(x)\varphi^{\prime }(x)dx=a_{0}(q,\psi ,\varphi )+a_{1}(q,\psi ,\varphi )$, where ***q*** ↦ *a*_0_(***q***, *ψ*, *φ*) and ***q*** ↦ *a*_1_(***q***, *ψ*, *φ*) are clearly linear in ***q*** for ***q*** ∈ *Q*. Hence the bilinear form *a*(***q***, ·, ·) is affine and continuous in ***q*** Thus, by Theorem 3.4, we have that the semigroup generated by *a*(***q***, ·, ·), {*e*^*A*(*q*)*t*^ : *t* ≥ 0} = {*T*(*t*, ***q***) : *t* ≥ 0}, is (Fréchet) differentiable in ***q*** for ***q*** ∈ *Q*. Denote the derivative in ***q*** and $\bar{q}$ acting on *δ****q*** ∈ *Q* by $T_{q}(t,\bar{q})\delta q$.

Moreover for *t* ∈ (0, *T*] we have for ***q***_1_, ***q***_2_ ∈ *Q* and line segment *S* = {*s****q***_1_ + (1 − *s*)***q***_2_ : 0 ≤ *s* ≤ 1},

(3.4) $${\left\|T\left(t,q_{1}\right)-T\left(t,q_{2}\right)\right\|}_{L\left(V^{*},V\right)}\leq {\left\|q_{1}-q_{2}\right\|}_{1}\underset{x\in Q}{\sup}{\left\|T_{q}(t,x)\right\|}_{L\left(Q,L\left(V^{*},V\right)\right)}\leq \tilde{C}{\left\|q_{1}-q_{2}\right\|}_{1}$$

with $\|\cdot {\|}_{L\left(V*,V\right)}$ the operator norm, where the first inequality follows from the Mean Value Theorem on Banach spaces (see Theorem 4 of Section 3.2 in [47]), and the second and third inequalities follow from the compactness of *Q*, Corollary 3.2, and the continuity of the map $T_{q}(t,\bar{q}))\mapsto \|T_{q}(t,\bar{q})){\|}_{L\left(Q,L\left(V*,V\right)\right)}$.

Further, under zero-order hold the differentiability and Lipschitz properties of $\hat{A}(q)=e^{A(q)\tau }=T(\tau ,q)$ remain. Considering $\hat{B}(q)$ from Section 2.1, $\hat{B}(q)=(I-\hat{A}(q))\left\{(0,\xi )-A{\left(q\right)}^{-1}\left(\frac{q_{3}q_{2}}{q_{1}},0\right)\right\}$, we find that it is a sum and product of ***q***-differentiable and ***q***-Lipschitz terms and thus is differentiable and Lipschitz in ***q***. Since *h* and *y* as in eq. (2.12) are a composition and sum of ***q***-differentiable terms they remain differentiable. Further, using eq. (3.4) we have the following Lipschitz bound for all *j* ∈ {0, 1, . . . , *K*} and ***q***, $\bar{q}$ in the interior of *Q*,

(3.5) $$\left|h_{j}(q)-h_{j}(\bar{q})\right|\leq \|\hat{C}{\|}_{L\left(H_{q},\mathbb{R}\right)}[{\left\|\hat{A}{\left(q\right)}^{j}-\hat{A}{\left(\bar{q}\right)}^{j}\right\|}_{L\left(H_{q}\right)}\|\hat{B}(q){\|}_{L\left(\mathbb{R},H_{q}\right)}+\;\;\;\;\;\;\;\;\;\;\;\;\;\;\;\;\;\;\;\;\;\;\;\;\;\;\;\;\;\;\;\;\;\;\;\;\;\;\;\;\;\;\;\;\;\;\;\;\;\;\;\;\;\;\;\;\;\;\;\;\;\;\;\;\;\;\;\;\;\;\;\;\;\;\;\;\;\;\;\;\;\;\;\;{\left\|\hat{A}{\left(\bar{q}\right)}^{j}\right\|}_{L\left(H_{q}\right)}\|\hat{B}(q)-\hat{B}(\bar{q}){\|}_{L\left(\mathbb{R},H_{q}\right)}]\;\;\;\;\;\;\;\;\;\;\;\;\;\;\;\;\;\;\;\;\;\;\;\;\;\;\;\;\leq C_{1}\left[{\left\|\hat{A}{\left(q\right)}^{j}-\hat{A}{\left(\bar{q}\right)}^{j}\right\|}_{L\left(H_{q}\right)}+\|\hat{B}(q)-\hat{B}(\bar{q}){\|}_{L\left(\mathbb{R},H_{q}\right)}\right]\;\;\;\;\;\;\;\;\;\;\;\;\;\;\;\;\;\;\;\;\;\;\;\;\;\;\;\leq C_{1}\left({\tilde{C}}_{\hat{A}}+{\tilde{C}}_{\hat{B}}\right){\left\|q_{1}-q_{2}\right\|}_{1}$$

for *C*_1_ the max of the operator norms for $\hat{A}(q)$, $\hat{B}(q)$, $\hat{C}$ over *Q*, and ${\tilde{C}}_{\hat{A}}$, ${\tilde{B}}_{\hat{A}}$ the max Lipschitz constants of $\hat{A}(q)$ and $\hat{B}(q)$ over all *k* and *Q*. The final inequality above follows from eq. (3.4) by noticing that for all $\hat{\varphi }\in H_{q}$, $\|\hat{A}(q)\hat{\varphi }-\hat{A}(\bar{q})\hat{\varphi }{\|}_{H_{q}}\leq C_{V}\|\hat{A}(q)\hat{\varphi }-\hat{A}(\bar{q})\hat{\varphi }{\|}_{V}$ and that by identification the supremum over V* is larger than that over *H*_***q***_. For ${\hat{A}}^{n}(q)$ and ${\hat{B}}^{n}(q)$ as in eq. (2.9) by a repetition of the above arguments we maintain differentiability in ***q***, and thus *h*^*n*^ and *y*^*n*^ as in eq. (2.12) are differentiable and Lipschitz in ***q***.

Lastly, for all *i* = 1, . . . , *R*, *j* = 1, . . . , *K* we have that for ***q***, $\bar{q}\in Q$

(3.6) $$\left|y_{j}^{i}(q)-y_{j}^{i}(\bar{q})\right|\leq \sum_{\ell =0}^{j-1}\left|h_{j-\ell -1}(q)-h_{j-\ell -1}(\bar{q})\right|u_{j}^{i}\leq {\tilde{M}}^{\left(K+1\right)}(K+1)\|q-\bar{q}{\|}_{1}$$

where $\left\{u_{j}^{i}\right\}$ are BrAC values bounded by definition to be in [0, 1], $\tilde{M}$ is the Lipschitz constant from eq. (3.5), and *K* + 1 is the fixed upper bound on the number of temporal observations, *j*. Hence, the Lipschitz constant for $y_{j}^{i}$ is independent of (*i*, *j*). By noticing that the previous statement holds for $y_{j}^{i,n}$ with a repetition of the work leading to eq. (3.6), our lemma has been proved. □

A direct consequence of Lemma 3.1 is that for all *i* = 1, 2, . . . , *R* with $K_{i}(\bar{q},q)$ and $\Lambda_{i}(\bar{q},q)$ as in the statement of Theorem 3.3, we have

(3.7) $$\left|K_{i}(\bar{q},q)\right|=\left|E_{\bar{q}}\left[\Lambda_{i}(\bar{q},q)\right]\right|\leq \sum_{j=0}^{K}\left|\frac{1}{2\sigma^{2}}\left[y_{j}^{i}{\left(\bar{q}\right)}^{2}-y_{j}^{i}(q)+2y_{j}^{i}(\bar{q})\left(y_{j}^{i}(q)-y_{j}^{i}(\bar{q})\right)\right]\right|\;\;\;\;\;\;\;\;\;\;\;=\frac{1}{2\sigma^{2}}\sum_{j=0}^{K}\left|{\left(y_{j}^{i}(\bar{q})-y_{j}^{i}(q)\right)}^{2}\right|\leq \frac{K{\bar{M}}^{2}}{2\sigma^{2}}\|\bar{q}-q{\|}_{1}=\tilde{\ell }\|\bar{q}-q{\|}_{1}$$

where *σ* > 0 is the standard deviation of the *N*(0, *σ*^2^) noise density, and $\bar{M}$ is the Lipschitz constant from eq. (3.6) that is independent of *i* (and *j*). Thus, for any δ* > 0, *i* ∈ {1, . . . , *R*}, and $\bar{q},\;q\in \{q\in Q:{\left\|q_{0}-q\right\|}_{1}<\delta *\}$, we have that ${\left\|f_{i,\bar{q}}-f_{i,q}\right\|}_{L_{1}}\leq {\left(2\left|K_{i}(\bar{q},q)\right|\right)}^{\frac{1}{2}}\leq {\left(2\tilde{\ell }\|\bar{q}-q{\|}_{1}\right)}^{\frac{1}{2}}<2{\left(\tilde{\ell }\delta *\right)}^{\frac{1}{2}}$ by the relationship between total variation and Kullback-Leibler distances, for $\tilde{\ell }$ as in eq. (3.7). If we let q* ∈ *Q* be such that ${\left\|f_{i,q_{0}}-f_{i,q^{*}}\right\|}_{L_{1}}>\delta^{*}$ and consider the set $G=\left\{q\in Q:{\left\|q*-q\right\|}_{1}<{\left(\delta *\right)}^{2}/(16\tilde{\ell })\right\}$, then G is strongly separated from ***q***_0_ (see Definition 3.1). This follows from the relationship between Affinity and total variation distance (via the Hellinger distance) as well as by noticing that for any density ν on G, the marginal density of ***V***^1^ satisfies ${\left\|f_{i,q^{*}}-v_{v}^{i}\left(V^{i}\right)\right\|}_{L_{1}}\leq \delta */2$ (for full example, see [32] or Example 3.5 in [42]). If this holds for all *i* then G and ***q***_0_ are strongly $\bar{\delta }$ separated with $\bar{\delta }$ independent of *i*.

With this example in mind we note that items 1 and 2 of Theorem 3.2 are satisfied if the following special condition is met:

1. For every δ* > 0, there exist sets *A*_1_, *A*_2_, . . . with *L*_1_ diameter less than δ*, $\mathrm{diam}\left(A_{i}\right)<\delta *,\;\underset{i\geq 1}{\cup }A_{i}=Q$, and $\sum_{i\geq 1}\sqrt{\pi_{0}\left(A_{i}\right)}<\infty$ for the mappings $q\mapsto f_{i,q}$

where *π*_0_ is the prior over *Q*. This follows from the fact that if special item 1 holds then we may take an ε*-neighborhood of ***q***_0_, $U=\left\{q\in Q:{\left\|f_{i,q_{0}}-f_{i,q}\right\|}_{L_{1}}<\varepsilon *\;\forall i\right\}$. As discussed above, since ${\left\|f_{i,\bar{q}}-f_{i,q}\right\|}_{L_{1}}$ is independent of *i*, *U* is non-empty and contains the set $\left\{q\in Q:{\left\|q_{0}-q\right\|}_{1}<{\left(\varepsilon *\right)}^{2}/(4\tilde{\ell })\right\}$. Now set $\delta *={\left(\varepsilon *\right)}^{2}/(16\tilde{\ell })$, and by compactness cover *Q* with a finite number of disjoint sets *A*_*i*_ determined by the balls $\left\{q\in Q:{\left\|{\bar{q}}_{i}-q\right\|}_{1}<\delta *\right\}$ with model ***q*** ↦ *f*_*i*,***q***_, where ${\left\{{\bar{q}}_{i}\right\}}_{i=1}^{\gamma }$ represents a finite set of points in *Q* chosen so that $\underset{i\geq 1}{\cup }A_{i}=Q$. From these *A*_*i*_’s we have that the finite subset that intersect with *U*^c^ must cover *U*^c^. This finite subset of *A*_*i*_’s subsequently satisfies the assumptions of Theorem 3.2. Specifically, the strong separation condition is satisfied as per the discussion leading up to special item 1 by noticing that on each *A*_*i*_ we have ${\left\|f_{i,q_{0}}-f_{i,{\bar{q}}_{i}}\right\|}_{1}>\varepsilon *$, and the convergent sum condition is satisfied by the fact that the *A*_*i*_’s can be considered (made) mutually exclusive with union contained in *Q*. We now state and prove our main theorem.

#### Theorem 3.5.

*For Q a rectangle in the interior of the positive orthant of ${\mathbb{R}}^{2}$, a prior π*_0_ *with compact support Q and a density that is continuous on Q, i.i.d noise distributed as N*(0, *σ*^2^) *for σ* > 0, *data* ${\left\{V^{i}\right\}}_{i=1}^{R}$ *drawn from independent but not identically distributed distributions generated by f*_i,**q**_ *as in* eq. (3.2), *Hilbert spaces H*_*q*_ *and V as in* eq. (2.4), *bilinear form* a(q,⋅,⋅):V×V→ℝ *as in* Section 2.1 *with q*_3_ *and q*_4_ *assumed known, induced infinitesimal generator A*(***q***) *as in Section 2.1, and true parameter* ***q***_0_ ∈ *Q, we have that our posterior π*(·|{***V***^*i*^}) *as in* eq. (3.2) *is consistent for* ***q***_0_ *as R* → ∞.

##### Proof.

For any set *A* ⊂ *Q* with ***q***_0_ ∉ *A* we will use the form of *π*(*A*|{***V***^*i*^}) as in eq. (3.2) and handle the numerator, *J*_*A*_ and denominator, *J* separately.

First, as *Q* is compact, for any *δ* > 0 we may cover *Q* by a finite number of sets *A*_*i*_, *i* = 1, 2, . . . , *γ* where each *A*_*i*_ is a subset of an *L*_1_ ball in *Q*. That is, for every *i* and ***q***, $\bar{q}\in A_{i}$ we have that $\|q-\bar{q}{\|}_{1}<\delta$. For *R* large enough, if on each *A*_*i*_ we consider the model ***q*** ↦ *f*_*i*,***q***_ for *i* ∈ {1, . . . , *R*} and *f*_*i*,***q***_ the density of the random variable $V^{i}$ with ***q*** assumed known, then special item 1 is satisfied for prior *π*_0_. Hence, by Theorem 3.2 we have that for some *β*_0_ > 0, $e^{R\beta_{0}}J_{A}\left(\left\{V^{i}\right\}\right)\to 0$ a.s. $P_{q_{0}}^{\infty }$ as *R* → ∞.

Now for Λ_*i*_, $K_{i}$, and *S*_*i*_ as in the statement of Theorem 3.3, for *i* = 1, 2, . . . , *R* and ***q*** ∈ *Q*, we have $\left|K_{i}\left(q_{0},q\right)\right|\leq \tilde{\ell }{\left\|q_{0}-q\right\|}_{1}$ and

$$S_{i}\left(q_{0},q\right)=\sum_{j=0}^{K}{\mathrm{Var}}_{q_{0}}\left[\frac{1}{2\sigma^{2}}\left(y_{j}^{i}{\left(q_{0}\right)}^{2}-y_{j}^{i}{\left(q\right)}^{2}+2V_{k}\left(y_{j}^{i}\left(q_{0}\right)-y_{j}^{i}(q)\right)\right)\right]\;\;\;\;\;\;\;\;\;\;\;\;\;\;=\sum_{j=0}^{K}\frac{1}{4\sigma^{4}}{\mathrm{Var}}_{q_{0}}\left[2V_{j}^{i}\left(y_{j}^{i}\left(q_{0}\right)-y_{j}^{i}(q)\right)\right]=\sum_{j=0}^{K}\frac{4\sigma^{2}}{4\sigma^{4}}{\left(y_{j}^{i}(q)-y_{j}^{i}\left(q_{0}\right)\right)}^{2}\;\;\;\;\;\;\;\;\;\leq \frac{{\tilde{\ell }}^{2}}{4}{\left\|q_{0}-q\right\|}_{1}^{2}$$

for $\tilde{\ell }$ as determined by eq. (3.7).

Thus, for ***q***_0_, ***q*** ∈ *Q* we find that $\sum_{i\geq 1}\frac{S_{i}\left(q_{0},q\right)}{i^{2}}\leq \left(\frac{{\tilde{\ell }}^{2}}{4}{\left\|q_{0}-q\right\|}_{1}^{2}\right)\sum_{i\geq 1}\frac{1}{i^{2}}<\infty$. Further, by the bounds above we have that for every *ε* > 0 and *i*, $\left\{q:\left|K_{i}\left(q_{0},q\right)\right|<\varepsilon \right\}$ is non-empty and our choice in such ***q*** does not depend on *i*. Hence the set $\left\{q:\left|K_{i}\left(q_{0},q\right)\right|<\varepsilon \;\forall i\right\}$ is non-empty. Thus, for *B* = *Q* we satisfy the assumptions of Theorem 3.3 and therefore find that **∀***β* > 0, *e*^*Rβ*^*J*({***V***^*i*^}) → ∞ a.s. $P_{q_{0}}^{\infty }$ as *k* → ∞.

So for any set *A* ⊂ *Q* with *q*_0_ ∉ *A*, from eq. (3.2) we have that *π*(*A*|{***V***^*i*^}) → 0 a.s. $P_{q_{0}}^{\infty }$ as *R* → ∞ and thus the theorem has been proved. □

From Lemma 3.1 we find that we maintain the differentiability and Lipschitz properties of the finite-dimensional semigroup as in eq. (2.9) and respective kernel as in eq. (2.12). Thus, with a straightforward rewriting of eq. (3.2) and eq. (3.2) in terms of the finite-dimensional posterior eq. (2.20), and repetition of the work following Lemma 3.1 through the proof of Theorem 3.5 we have the following corollary.

#### Corollary 3.3.

*Under the same hypotheses as Theorem 3.5, for fixed positive integer n we have that our finite-dimensional posterior π*^*n*^(·|{***V***^*i*^}) *as in* eq. (2.20) *is consistent at* ***q***_0_ *as R* → ∞.

### 3.3. Deconvolution of BrAC from TAC

In this section we consider the problem of using the biosensor measured TAC signal to estimate BrAC. We do this by deconvolving it; to wit we invert the convolution given in eq. (2.12) subject to a positivity constraint and regularization to mitigate the inherent ill-posedness of the inversion. Recall that the convolution given in eq. (2.12) was found by solving the finite-dimensional discrete time system eq. (2.10) derived from eq. (2.2). We employ the method originally described in [25], wherein the problem is formulated as a constrained, regularized, optimization problem (see, for example, [48]).

We first briefly summarize the treatment in [25] and then follow by showing how our work is able to make direct use of this theory. Let $\tilde{V}$ and $\tilde{H}$ be Hilbert spaces forming a Gelfand Triple, $\tilde{V}↪\tilde{H}↪{\tilde{V}}^{*}$. For an admissible set *Q*, a compact subset of the positive orthant of ${\mathbb{R}}^{2}$, with ***q*** ∈ *Q*, let *A*(***q***) be an abstract parabolic operator defined by a sesquilinear form a(q,⋅,⋅):V×V→ℝ (i.e., one that satisfies items 1 to 3 in Section 2.1) that when restricted to $\left\{\varphi \in \tilde{V}:A(q)\varphi \in \tilde{H}\right\}$ generates a holomorphic semigroup on $\tilde{H},\left\{e^{A(q)t}:t\geq 0\right\}$. For bounded operators $B(q)\in L(\mathbb{R},\tilde{H})$ and $C(q)\in L(\tilde{H},\mathbb{R})$ consider the input/output system where $\dot{x}(t)=A(q)x(t)+B(q)u(t)$, $x(0)=x_{0}\in \tilde{H}$, and *y*(*t*) = *C*(***q***)*x*(*t*) where on the interval [0, *T*], *u* ∈ *L*_2_(0, *T*) is the input, *y* the output, and *x* is the state variable.

For sampling interval *τ* > 0 and zero-order hold input *u*(*t*) = *u*_*j*_, *t* ∈ [*jτ*, (*j* + 1)*τ*), *j* = 0, 1, 2, . . . the corresponding sampled-time system is given by

(3.8) $$x_{j+1}=\hat{A}(q)x_{j}+\hat{B}(q)u_{j}\;x_{0}\in \tilde{H},\;\;\;\;\;\;\;y_{j}=\hat{C}(q)x_{j}$$

where $\hat{A}(q)=e^{A(q)\tau }\in L(\tilde{H},\tilde{H})$, $\hat{B}(q)=\int_{0}^{\tau }e^{A(q)s}B(q)ds\in L(\mathbb{R},\tilde{H})$ and $\hat{C}(q)=C(q)\in L(\tilde{H},\mathbb{R})$, $x_{j}=x(j\tau )\in \tilde{H}$, $y_{j}=y(j\tau )\in \mathbb{R}$ *j* = 0, 1, 2, . . . , and for all *j*, $\left\{u_{j}\right\}\subset \mathbb{R}$ are zero-order hold input values.

Now let q be a random variable with support the parameter space *Q*. For $\tilde{\pi }$ the probability measure of q, define the Bochner spaces $V=L_{\tilde{\pi }}^{2}(Q;\tilde{V})$, $H=L_{\tilde{\pi }}^{2}(Q;\tilde{H})$, and $U=L_{\tilde{\pi }}^{2}(Q;\mathbb{R})$. It is easily shown that the spaces V and H form a Gelfand triple $V↪H↪V^{*}$. Define a(⋅,⋅):V×V→ℝ by $a(\varphi ,\psi )=E_{\tilde{\pi }}[a(q,\varphi (q),\psi (q))]=\int_{Q}a(Q,\varphi (q),\psi (q))d\tilde{\pi }(q)$ for φ,ψ∈V. Then, as in Section 2.1, the form −a(⋅,⋅) satisfies items 1 to 3 and therefore defines a linear map A that when restricted to {φ∈V:Aφ∈H}, generates an analytic semigroup on H, $\left\{e^{At}:t\geq 0\right\}$.

Assume further that the map ***q*** ↦ *B*(***q***) is in $L_{\infty }(Q,L(\mathbb{R},\tilde{H}))$ and that the map ***q*** ↦ *C*(***q***) is in $L_{2}(Q,L(\tilde{H},\mathbb{R}))$ with respect to the measure $\tilde{\pi }$. (Note that since the domain space in $L(\mathbb{R},\tilde{H})$ and the co-domain space in $L(\tilde{H},\mathbb{R})$ are both ℝ, it follows that in fact the mapping $q\mapsto B(q)\in L_{\infty }(Q,\tilde{H})$, and by the Riesz Representation Theorem, that effectively the mapping $q\mapsto C(q)\in L_{2}(Q,\tilde{H})=H$). Then define bounded linear operators B∈L(U,H) and C∈L(H,ℝ) by ${\langle Bu,\psi \rangle }_{H}=E_{\tilde{\pi }}\left[{\langle B(q)u(q),\psi (q)\rangle }_{\tilde{H}}\right]=\int_{Q}\langle B(q)u(q),\psi (q)\rangle_{\tilde{H}}d\tilde{\pi }(q)$ and $C\psi =E_{\tilde{\pi }}[C(q)\psi (q)]=\int_{Q}C(q)\psi (q)d\tilde{\pi }(q)$, respectively, for u∈U and ψ∈H. It can then be shown [49,50] that for $u\in L_{2}([0,T],U)$ the solution to the input/output system from above with u(t)=u(t,q)=u(t) agrees with the solution to the system where $\dot{x}(t)=Ax(t)+Bu(t)$, $x(0)=x_{0}\in H$, and y(t)=Cx(t) for $\tilde{\pi }$—almost every ***q*** ∈ *Q*. Then with sampling interval *τ* > 0 as in eq. (3.8), and zero-order hold input $u(t)=u_{j},t\in [j\tau ,(j+1)\tau )$, $u_{j}\in U$, *j* = 0, 1, 2, . . . , this system becomes

(3.9) $$x_{j+1}=\hat{A}x_{j}+\hat{B}u_{j}\;x_{0}\in H\;\;\;\;\;\;\;y_{j}=\hat{C}x_{j}$$

for *j* = 0, 1, 2, . . . where $\hat{A}=e^{A\tau }\in L(H,H)$, $\hat{B}=\int_{0}^{\tau }e^{As}Bds\in L(U,H)$, and $\hat{C}\in L(H,\mathbb{R})$.

Now, with eq. (3.9), note that $\left\{u_{j}\right\}\subset U$ is obtained by zero-order hold sampling a continuous time signal. That is, the input to eq. (3.9) is $u(j\tau )=u_{j}\in U$ with u at least continuous on [0, *T*]. We seek an estimate for the input based on this model, wherein the input estimate u is a function of both time and the random parameters q. For optimization purposes (more precisely, to be able to include regularization) we require additional regularity. Given the time interval [0, *T*], let $u\in S(0,T)=H^{1}(0,T;U)$ and let U be a compact subset of *S*(0, *T*).

The input estimation or deconvolution problem is then given by

(3.10) $$u^{*}=\underset{U}{\arg\;\min}J(u)=\underset{U}{\arg\;\min}\sum_{k=1}^{K}{\left|y_{k}(u)-{\hat{y}}_{k}\right|}^{2}+\|u{\|}_{S(0,T)}^{2}$$

where $\|\cdot {\|}_{S(0,T)}^{2}$ is a norm on *S*(0, *T*) that will be defined below, $\left\{{\hat{y}}_{k}\right\}$ are measured output values, the term $\|u{\|}_{S(0,T)}^{2}$ serves as regularization, and $y_{k}(u)=\sum_{j=0}^{k-1}{\langle h_{k-j-1},u_{j}\rangle }_{U}$, for *k* = 1, 2, . . . , *K* with $u_{j}=u(j\tau )$ for *j* = 1, 2, . . . , *K* the zero-order hold input to the discrete time system eq. (3.9), and convolution filter $h_{\ell }=\hat{C}{\hat{A}}^{\ell -1}\hat{B}\in L(U,\mathbb{R})=U*$ (which is equal to U, by the Riesz Representation Theorem) where $\hat{C}\in L(H,\mathbb{R})$, $\hat{A}\in L(H,H)$, and $\hat{B}=\int_{Q}e^{As}Bds\in L(U,H)$.

Solving eq. (3.10) requires finite dimensional approximations. For index *M*, let $U^{M}$ define an approximating family of closed subsets of U, where each subset is contained within a corresponding finite dimensional subspace, $S^{M}$ of *S*(0, *T*). Further we require that for each u∈U there exists a sequence $\left\{u^{M}\right\}$ with $u^{M}\in U^{M}$ such that $u^{M}\to u$ in *S*(0, *T*) as *M* → ∞. For index *N*, let $V^{N}$ be an element of an approximating family of finite-dimensional subspaces of V, and let $P_{H}^{N}:H\to V^{N}$ be the orthogonal projection of H onto $V^{N}$. We also require of the spaces $V^{N}$ that for each v∈V, $P_{H}^{N}v\to v$ in V as *N* → ∞.

We next specify finite-dimensional operators ${\hat{A}}^{N}\in L\left(V^{N},V^{N}\right)$, ${\hat{B}}^{N}\in L\left(U,V^{N}\right)$, and ${\hat{C}}^{N}\in L\left(V^{N},\mathbb{R}\right)$ that define the finite-dimensional system analogous to eq. (3.9). That is, let $A^{N}:V^{N}\to V^{N}$ be given by ${\langle A^{N}\varphi^{N},\psi^{N}\rangle }_{H}=-a\left(\varphi^{N},\psi^{N}\right)$ for $\varphi^{N},\psi^{N}\in V^{N}$, ${\hat{A}}^{N}=e^{A^{N}\tau }$, $B^{N}=P_{H}^{N}B$, ${\hat{B}}^{N}=\int_{0}^{\tau }e^{A^{N}s}B^{N}ds$, and ${\hat{C}}^{N}=C$. In this way we obtain a doubly-indexed sequence of approximating finite-dimensional optimization or deconvolution problems given by

(3.11) $$u_{L}^{*}=\underset{U^{M}}{\arg\;\min}J^{L}(u)=\underset{U^{M}}{\arg\;\min}\sum_{k=1}^{K}{\left|y_{k}^{N}(u)-{\hat{y}}_{k}\right|}^{2}+\|u{\|}_{S(0,T)}^{2}$$

where *L* = (*M*, *N*), $h_{\ell }^{N}={\hat{C}}^{N}{\left({\hat{A}}^{N}\right)}^{\ell }{\hat{B}}^{N}\in U$, and

(3.12) $$y_{k}^{N}(u)=\sum_{j=0}^{k-1}\langle h_{k-j-1}^{N},u_{j}\rangle U,\;k=1,2,\ldots ,K.$$

Using the approximation properties of the subspaces $V^{N}$ and $U^{M}$ (that is, that for each $u\in U^{M}$ there exists a sequence $\left\{u^{M}\right\}$ with $u^{M}\in U^{M}$ and ${\left\|u^{M}-u\right\|}_{S(0,T)}\to 0$ as *M* → ∞, and that for each v∈V, ${\left\|P_{H}^{N}v-v\right\|}_{V}\to 0$ as *N* → ∞), and the corresponding operators ${\hat{A}}^{N}\in L\left(V^{N},V^{N}\right)$, ${\hat{B}}^{N}\in L\left(U,V^{N}\right)$, and ${\hat{C}}^{N}\in L\left(V^{N},\mathbb{R}\right)$, it can be shown that 1) for each multi-index *L*, eq. (3.11) admits a solution $u_{L}^{*}$, and 2) there exists a subsequence of $\left\{u_{L}^{*}\right\}$, $\left\{u_{L_{k}}^{*}\right\}\subset \left\{u_{L}^{*}\right\}$ with $u_{L_{k}}^{*}\to u*$ strongly as *k* → ∞ with u* a solution of eq. (3.10). Further, if in addition U is assumed to be a closed and convex subset of *S*(0, *T*), for each *M*, $U^{M}$ is a closed and convex subset of U, and the optimization problem given in eq. (3.10) admits a unique (with respect to sampling) solution, then the sequence of solutions to eq. (3.11), $\left\{u_{L}^{*}\right\}$ converges strongly, or in *S*(0, *T*) to the unique solution of eq. (3.10), u*. For the proofs of these results see Section 5 of [25]

To numerically carry out the requisite computations to actually determine $u_{L}^{*}$ for given values of *M*, *N* and *L* = (*M*, *N*), we continue to apply the results in [25] while also connecting them to our treatment in Sections 2.1 and 2.2 above. We assume that the feasible parameter set *Q* is a compact rectangle in the positive orthant of ${\mathbb{R}}^{2}$, we set $\tilde{H}=H_{q}$ and $\tilde{V}=V$ as in eq. (2.4), and we identify the operators in eq. (3.8) with those in eq. (2.6). Our distribution over q, $\tilde{\pi }$, is the finite-dimensional posterior $\pi^{n}\left(\cdot |\left\{V_{j}^{i}\right\}\right)$ for fixed *n* as in eq. (2.20) and we proceed with the Bochner spaces $V=L_{\pi^{n}\left(\cdot |V_{j}^{i}\right))}^{2}(Q;V)$ and $H=L_{\pi^{n}\left(\cdot \left|K_{j}^{i}\right|\right)}^{2}\left(q;H_{Q}\right)$ to achieve eq. (3.9).

For the state variables *x*_*j*_(*η*, ***q***) we have that *η* ∈ [0, 1] and ***q*** ∈ *Q* = [*a*_1_, *b*_1_] × [*a*_2_, *b*_2_] for 0 < *a*_*i*_ < *b*_*i*_, *i* = 1, 2. Further, for the inputs u(t,q) we have that *t* ∈ [0, *T*] and ***q*** ∈ *Q*. Let *n* be as in eq. (2.8) and *m* a positive integer, and we discretize [0, 1] and [0, *T*] using the sets of linear B-splines, ${\left\{\psi_{j}^{n}\right\}}_{j=0}^{n}$ and ${\left\{\zeta_{j}^{m}\right\}}_{j=0}^{m}$, respectively, on the uniform meshes, ${\left\{\frac{j}{n}\right\}}_{j=0}^{n}$ and ${\left\{\frac{iT}{m}\right\}}_{j=0}^{m}$, respectively. Further, for positive integers *m*_1_ and *m*_2_, we discretize *Q* with the 0^th^-order B-splines ${\left\{\chi_{i,j}^{m_{i}}\right\}}_{j=1}^{m_{i}}$, *i* = 1,2 defined with respect to the uniform grids ${\left\{a_{i}-\frac{\left(b_{i}-a_{i}\right)j}{m_{i}}\right\}}_{j=0}^{m_{i}}$, on [*a*_*i*_, *b*_*i*_], *i* = 1, 2.

Then for multi-indices *N* = (*n*, *m*_1_, *m*_2_) and *M* = (*m*, *m*_1_, *m*_2_) we define the approximating subspaces $V^{N}$ and $S^{M}$ as follows using tensor products. That is, let $V^{N}=\mathrm{span}{\left\{{\hat{\psi }}_{i}^{N}\right\}}_{i=1}^{N}=\mathrm{span}{\left\{\left(\psi_{j}^{n}(0)\chi_{1,j_{1}}^{m_{1}}\chi_{2,j_{2}}^{m_{2}},\psi_{j}^{n}\chi_{1,j_{1}}^{m_{1}}\chi_{2,j_{2}}^{m_{2}}\right)\right\}}_{j=0,j_{1}=1,j_{2}=1}^{n,m_{1},m_{2}}$, and $S^{M}=\mathrm{span}\{{\left(\zeta_{j}^{m}\chi_{1,j_{1}}^{m_{1}}\chi_{2,j_{2}}^{m_{2}}\right\}}_{j=0,j_{1}=1,j_{2}=1}^{m,m_{1},m_{2}}=\mathrm{span}{\left\{{\hat{\zeta }}_{i}^{M}\right\}}_{i=1}^{M}$, where $N=(n+1)m_{1}m_{2}$ and $M=(m+1)m_{1}m_{2}$. Standard approximation theoretic arguments (see, for example, [33]) can be used to argue that the subspaces defined in above satisfy the required approximation assumptions on $V^{N}$ and $S^{M}$. Then $x^{N}\in V^{N}$ and $u^{M}\in S^{M}$, can be written as $x^{N}(\eta ;q)=\sum_{i=0,i_{1}=1,i_{2}=1}^{n,m_{1},m_{2}}x_{i,i_{1},i_{2}}^{N}\psi_{i}^{n}(\eta )\chi_{1,i_{1}}^{m_{1}}\left(q_{1}\right)\chi_{2,i_{2}}^{m_{2}}\left(q_{2}\right)$ and $u^{M}(t;q)=\sum_{i=0,i_{1}=1,i_{2}=1}^{m,m_{1},m_{2}}u_{i,i_{1},i_{2}}^{M}\zeta_{i}^{m}(t)\chi_{1,i_{1}}^{m_{1}}\left(q_{1}\right)\chi_{2,i_{2}}^{m_{2}}\left(q_{2}\right)$, respectively.

Then with the bases for $V^{N}$ and $S^{M}$ as chosen above, it is an elementary exercise to determine the matrix representations for the operators $A^{N}\;$, $B^{N}$, $C^{N}$, ${\hat{A}}^{N}$, ${\hat{B}}^{N}$ and ${\hat{C}}^{N}$. It then follows that eq. (3.9) takes a matrix system where for *k* = 1, 2, . . . , *K*, $M^{N}X_{k+1}^{N}=K^{N}X_{k}^{N}+B^{L}U_{k}^{N}$ and $y_{k}^{L}={\mathbb{C}}^{N}X_{k}^{N}$ with $X_{k}^{N}\in {\mathbb{R}}^{N}$ the coefficients of the basis elements $\left\{{\hat{\psi }}_{i}^{N}\right\}$, $U_{k}^{N}\in {\mathbb{R}}^{M}$ the coefficients of the basis elements $\left\{{\hat{\zeta }}_{i}^{M}\right\}$ as in the previously mentioned approximating subspaces, $M^{N}\in {\mathbb{R}}^{N\times N}$ a matrix with entries ${\left[M^{N}\right]}_{i,j}={\langle {\hat{\psi }}_{i},{\hat{\psi }}_{j}\rangle }_{H}$, $K^{N}\in {\mathbb{R}}^{N\times N}$ a matrix with entries ${\left[K^{N}\right]}_{i,j}={\langle A^{N}{\hat{\psi }}_{i}^{N},{\hat{\psi }}_{j}^{N}\rangle }_{H}$, $B^{L}\in {\mathbb{R}}^{N\times M}$ a matrix with entries ${\langle B^{N}{\hat{\zeta }}_{i}^{M},{\hat{\psi }}_{j}^{N}\rangle }_{H}$, and ${\mathbb{C}}^{N}\in {\mathbb{R}}^{1\times M}$ given by [1, 0, . . . , 0]. From here the matrix representation of $h_{k}^{L}$ (with *L* = (*M*, *N*) in place of *N* due to the joint dependence on the multi-indices *M* and *N*) can be found using this matrix system.

We note that the optimization problem eq. (3.11) is a constrained problem, in that $U_{k}^{N}$ of the previously stated matrix system are to be non-negative. With a proper placement of $\left\{h_{k}^{L}\right\}$ into the block matrix ${ℍ}^{L}$, the approximating deconvolution problem eq. (3.11) is now given by $u_{L}^{*}=\underset{U^{M}}{\arg\;\min}J^{L}(u)$ where we have that

(3.13) $$J^{L}\left(u;r_{1},r_{2}\right)={{\left\|\left[\begin{array}{c} {ℍ}^{L}\\ {\left(r_{1}{\mathbb{Q}}_{1}^{M}+r_{2}{\mathbb{Q}}_{2}^{M}\right)}^{\frac{1}{2}} \end{array}\right]U^{M}-Y^{M}\right\|}^{2}|}_{{\mathbb{R}}^{K+K.M}},$$

where $U^{M}$ is the KM dimensional column vector of the coefficients of $u\in U^{M}$, and $Y^{M}$ is the K+KM column vector of measured output values $\left\{{\hat{y}}_{k}\right\}$ followed by KM zeros. Further, ${\mathbb{Q}}_{i}^{M}$ for *i* = 1, 2 are matrices with entries given by the U inner products of the basis elements for the subspaces $S^{M}$ as determined by the regularization term $\|u{\|}_{S(0,T)}^{2}$ below. Note that the regularization term $\|u{\|}_{S(0,T)}^{2}$ is derived from a weighted inner product on *S*[0, *T*] and thus corresponds to a squared norm on *S*(0, *T*) given by $\|u{\|}_{S(0,T)}^{2}=r_{1}\int_{0}^{T}\|u(t){\|}_{U}^{2}dt+r_{2}\int_{0}^{T}\|\dot{u}(t){\|}_{U}^{2}dt$.

The values of the regularization weights used in eq. (3.13), $r_{i}=r_{i}^{*}>0$ for *i* = 1, 2 are chosen optimally. Indeed, in order to find $\left(r_{1}^{*},r_{2}^{*}\right)$, BrAC-TAC input-output training data pairs, ${\left\{\left(u_{j}^{i},V_{j}^{i}\right)\right\}}_{i=0,j=0}^{P,K}$ are used to optimize (*r*_1_, *r*_2_) via the following scheme:

(3.14) $$\left(r_{1}^{*},r_{2}^{*}\right)=\underset{\left(r_{1},r_{2}\right)\in {\mathbb{R}}^{+}\times {\mathbb{R}}^{+}}{\arg\;\min}\sum_{i=1}^{R}\sum_{j=1}^{K}\left({\left|{\bar{u}}_{L;j-1}^{i,*}-u_{j-1}^{i}\right|}^{2}+{\left|{\tilde{y}}_{L;j}^{i,*}-V_{j}^{i}\right|}^{2}\right)$$

where ${\bar{u}}_{L;j}^{i,*}=E_{\tilde{\pi }\left(\theta^{*}\right)}\left[{\tilde{u}}_{L;j}^{i,*}\right]$, ${\tilde{u}}_{L;j}^{i,*}$ are the predicted BrAC values found by finding the minimum of *J*^*L*^( · ; *r*_1_, *r*_2_) given by eq. (3.13) with *r*_1_ and *r*_2_ candidate values for the regularization weights from a specified feasible set in the positive orthant of ${\mathbb{R}}^{2}\;$, ${\mathbb{R}}^{+}\times {\mathbb{R}}^{+}$ and ${\tilde{y}}_{L;j}^{i,*}$ are the TAC values found by using ${\tilde{u}}_{L;j}^{i,*}$ as input to eq. (3.12).

### 3.4. Numerical results

All of the data used in the studies detailed below, unless otherwise specifically stated (e.g., as in Section 3.4.2), were collected in USC IRB approved human subject experiments designed and run by researchers in the laboratory of one of the authors (S. E. L.) as part of a National Institutes of Health (NIH) funded investigation (see, [51]). These experiments were carried out in controlled environments wherein 40 participants completed one to four drinking episodes, with viable data recorded in 146 drinking episodes. BrAC was obtained using Alco-sensor IV breath analyzer devices from Intoximeters, Inc, St. Louis, MO, and participants each wore two SCRAM (Secure Continuous Remote Alcohol Monitoring) devices manufactured by Alcohol Monitoring Systems (AMS) in Littleton, Colorado (see Figure 1) simultaneously placed on the participants’ left and right arms for TAC. For each separate SCRAM device, participants started their readings with a TAC and BrAC of 0.000, consumed alcohol (equivalent across all sessions per participant) in one of three different drinking patterns (single: over 15 minutes; dual: over two 15-min periods spaced 30-minutes apart; or steady: over 60 minutes), and then ended their session when their TAC and BrAC had returned to 0.000. We note that the placement of the two sensors challenges the independence assumption from Section 2.2, but for experimental purposes we will include all of the data as independently measured drinking episodes with this caveat in mind. In addition, we did not focus on any specific drinking pattern as including all possible patterns is in line with real-world, varying drinking patterns and may improve the generalizability of our model. In the calculations of Sections 3.4.1 and 3.4.2, as in eq. (2.5), time is discretized by a constant sampling time *τ* of 5 minutes and is subject to our zero-order hold assumption. While this challenges the implications of our zero order hold assumption, namely that *τ* = .0833 hours implies that subjects’ BAC is constant for 5 minutes, this restriction is needed as computational complexity becomes unstable as *τ* decreases. In order to achieve this sampling time, we first linearly interpolate all of the data (both BrAC and TAC), and then re-sample at our desired rate of *τ* = 5. For Section 3.4.3, a *τ* will be discussed. Further, in all sections we assume a truncated multivariate normal (tMVN) prior *π*_0_ (as in eq. (2.20)) on ***q*** with mean *μ* and covariance matrix Σ which varies from example to example.

Unfortunately, the USC IRB approved experiments for collecting human subject data were not designed around the problem of estimating the sensor collection chamber inflow and outflow parameters, *q*_3_ and *q*_4_ as in eq. (2.3), nor do the authors have the laboratory facilities or expertise to determine them experimentally. Moreover, since the approach developed in sections 2.1 and 2.2, and in particular our underlying hybrid PDE/ODE model given in 2.3, are relatively novel, no values for *q*_3_ and *q*_4_ are available from either the manufacturers of the sensors or the current literature. Consequently, for the purposes of this study we have chosen values for *q*_3_ and *q*_4_ arbitrarily as *q*_3_ = *q*_4_ = 1. However, we note that the precise values chosen for *q*_3_ and *q*_4_ had no perceptible qualitative effect on the results to be presented below. Finally we note that all computational work was done in Python 3.7.2 and includes ported MATLAB code from the work of [15,21,24,25], in particular with respect to the creation of the finite-dimensional, discrete-time kernel as in eq. (2.12). Ported code was verified against the original code through the use of unit tests.

#### 3.4.1. Convergence in distribution

**Figure 2.**
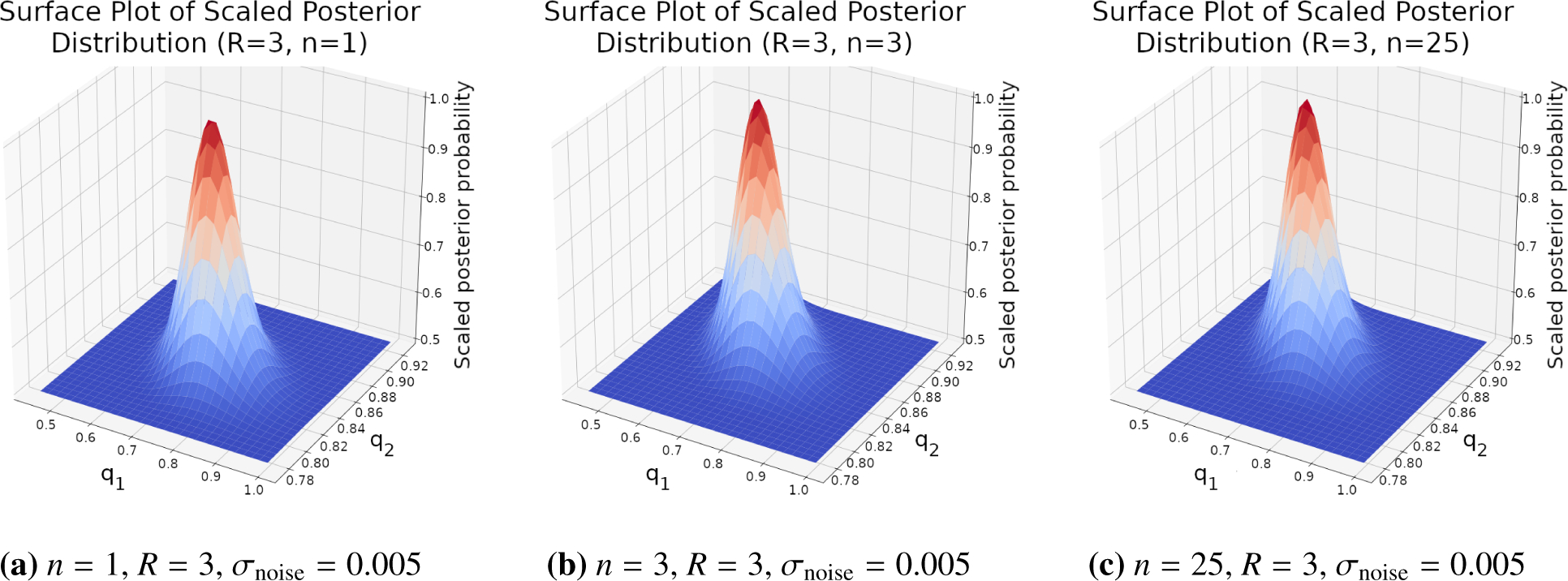
Posterior distribution surface plots for varying finite dimensional approximations of the kernel from eq. (2.12).

**Figure 3.**
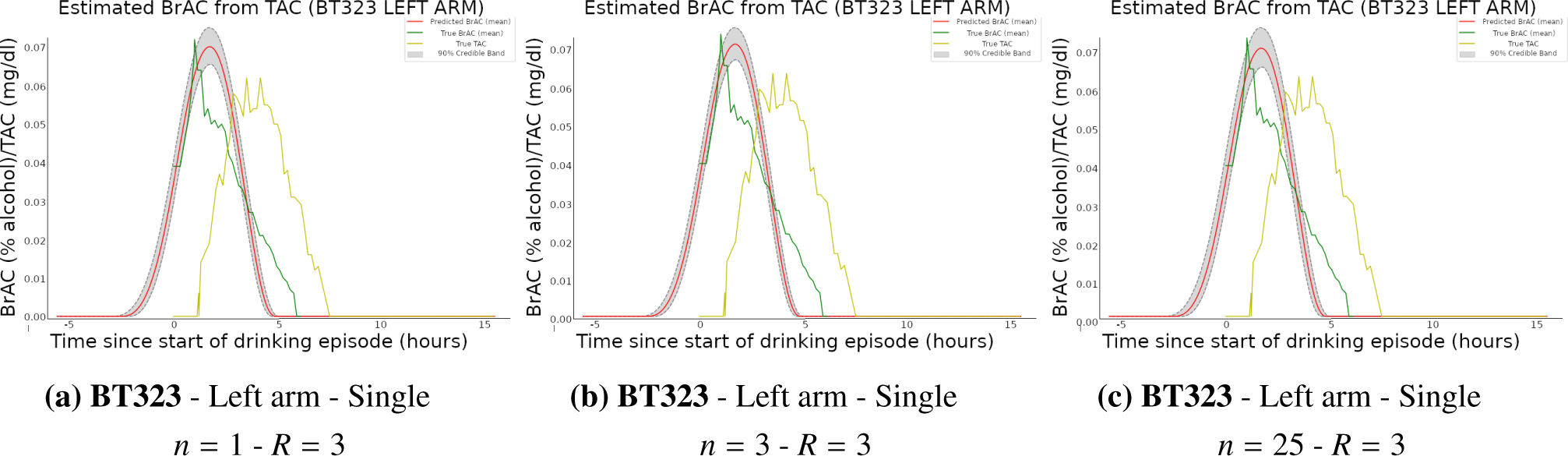
Deconvolutions for differing approximating dimension values, *n*, associated with posteriors from Figure 2 and Table 1.

We used surface plots as well as Metropolis Hastings (MH) Markov Chain Monte Carlo (MCMC) methods to validate our convergence in distribution results. Throughout the results described here we have that from eq. (2.14) for all sample times the i.i.d. noise *ε* is distributed as *N*(0, 0.005^2^), and prior *π*_0_ as in eq. (2.20) is distributed as the optimal distribution found in Section 6 of [25]. Specifically, the prior is a tMVN random variable with mean, $\mu =\left(\begin{array}{l} 0.6318\\ 1.0295 \end{array}\right)$ and covariance matrix, $\Sigma =\left(\begin{array}{ll} 0.0259 & 0.0077\\ 0.0077 & 0.1232 \end{array}\right)$ with the feasible parameter set, *Q*, taken to be *Q* = [0.01, 2.2877] × [0.01, 2.1410] for ***q*** = [*q*_1_, *q*_2_]^*T*^. The choice of 0.005 for the standard deviation of *ε* was made to limit the role of noise in our subsequent sampling algorithms so that we may focus on the role of the dimension of our approximating system in the resulting posterior distribution. In addition, when comparing this choice in standard deviation to the peak TAC values of our training dataset, we had a typical peak TAC to noise ratio of 20. For computational reasons, we limit ourselves to measurements from a random subgroup of *R* = 3 subject drinking episode measurements. Figure 2 contains the resulting surface plots for *n* values of 1, 3, and 25. Further, Table 1 contains the means and credible regions for *n* values of 1, 2, 3, and 25 as determined by respective 1000 sample (1100 draws with a 100 draw burn-in period) MH MCMC sampling runs. The MCMC sample size chosen here was due to computational complexities and runtimes. Figure 3 displays deconvolution results for a randomly chosen, non-training drinking episode for different values for the dimension of the approximating system, *n*. This figure used the method from Section 3.3 along with the resulting posteriors as shown in Figure 2 and Table 1.

#### 3.4.2. Consistency

We again used surface plots as well as MH MCMC sampling methods to verify our consistency results. For these studies we have assumed that the noise *ε* is now distributed as *N*(0, 0.025^2^) while our prior *π*_0_ from eq. (2.20) is still the optimal distribution found in Section 6 of [25]. That is, *π*_0_ is a tMVN with $\mu =\left(\begin{array}{l} 0.6318\\ 1.0295 \end{array}\right)$ and covariance matrix, $\Sigma =\left(\begin{array}{ll} 0.0259 & 0.0077\\ 0.0077 & 0.1232 \end{array}\right)$ with bounds [0.01, 2.2877] and [0.01, 2.1410] for *q*_1_ and *q*_2_, respectively. The choice for a distribution for the random noise is meant to simulate a more realistic situation where little is assumed known about any external effects that play a role in perturbing the sensor measurements. Consequently the data is assumed noisy. When comparing this choice for the standard deviation of the noise process to the peak TAC values of our training dataset, we had a typical peak TAC to noise ratio of 8.

To test Theorem 3.5, we generated 276 TAC values using subject-measured BrAC values via eq. (2.14) with a predetermined ***q***_0_ value of [1, 1], *n* = 24, and noise variance of 0.025^2^. Table 2 displays the calculated means and 90% credible circle radii for the posterior distribution eq. (2.22) for increasing amounts of idealized (BrAC, TAC) data pairs (*R*) all generated using the “true” ***q***_0_ value previously stated. To calculate these values, MH MCMC samples were drawn with a sample of size 1400 (1500 data points with a 100 sample burn-in phase) where the MCMC sample size was increased from that of Section 3.4.1 due to the increase in noise variance.

**Figure 4.**
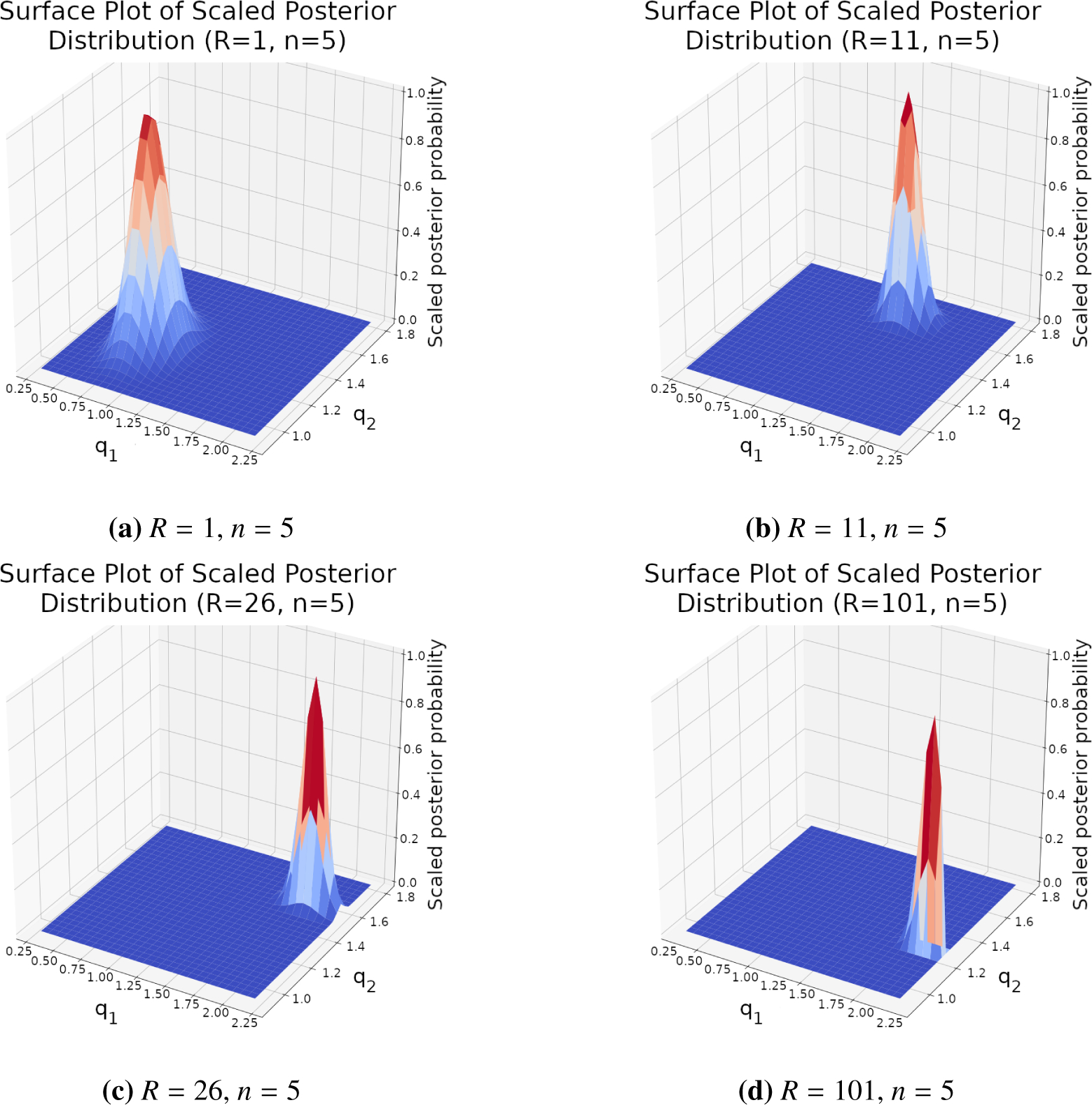
Posterior distribution surface plots for varying amounts of collected data, *m*. All images use noise and prior distributions as stated in Section 3.4.2.

We now investigate the results of Section 3.2 with respect to the field-measured (BrAC, TAC) data pairs. Note that we no longer are able to know the true value of the parameters, ***q***_0_. Surface plots for increasing amounts of subject drinking episode measurements, *R* = 1, 26, 76, and 101 are contained within Figure 4. Table 3 displays the calculated means and 90% credible circle radii for increasing numbers of subjects, and thus data (corresponding to *R* as in Section 3.2) included in determination of the prior. To calculate these values, for each *R*, we again used 1400 MH MCMC samples (1500 draws with a 100 sample burn-in phase) generated according to our chosen prior.

#### 3.4.3. Deconvolution

As in Section 3.3, we rely on the treatment in [25] for deconvolving BrAC from TAC using a distribution for q over *Q*. The chosen distribution was the posterior eq. (2.22) with *n* = 3. To determine the posterior we elected to investigate the case where a non-informative, or what is more aptly described as an uneducated, prior was used. Thus we chose a prior of a tMVN random variable with bounds [0.01, 10] × [0.01, 10], and parameters $\mu =\left(\begin{array}{l} 5\\ 5 \end{array}\right)$ and $\Sigma =\left(\begin{array}{cc} 0.7 & 0.1\\ 0.1 & 0.55 \end{array}\right)$. The noise used was distributed as $N\left(0,0.025^{2}\right)$. Note that this choice in prior also highlights the effects of data on the posterior by not providing any initial information to the posterior. When comparing this choice in noise standard deviation to the peak TAC values of our training dataset, we had a typical peak TAC to noise ratio of 8. Further, for the subspaces from Section 3.3 we set our discretization to be *n* = 3, time discretization as *m* = 1300, and discretized *Q* with *m*_1_ = *m*_2_ = 20. As with Section 3.4.2, the noise distribution is meant to simulate a situation where little is known about external effects that play a role in determining noise, and so the data is assumed noisy.

In all of the numerical results presented and discussed in this section, the test dataset used consisted of five drinking episodes from four different participants. These drinking episodes were chosen heuristically so that the test dataset had two drinking episodes with peak BrAC greater than peak TAC, two drinking episodes with peak BrAC less than peak TAC, and one drinking episode with peak BrAC within 0.015 of peak TAC (deemed, “close”). The remaining drinking episodes were used as training data with the added restriction that whenever the desired number of training sets to be used was not too large, BrAC/TAC pairs from any participant who had a dataset included in the selected test data, would be excluded from being among the data used for determining the posterior. The primary exception to this restriction being Figure 6c, wherein we allowed all data that wasn’t the current test data point to be included in the training set.

**Figure 5.**
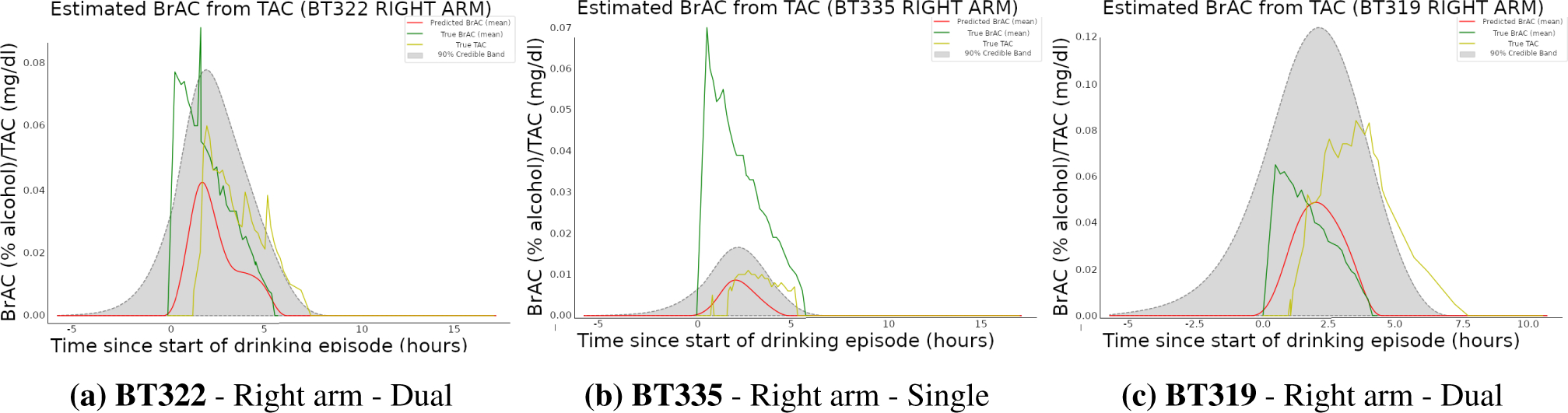
BrAC deconvolution given TAC and predicted ***q*** values for varying test data participants’ right arm data with conservative credible regions shaded in gray. Across subfigures, all training data remained constant with *R* = 25. Prior used was tMVN with bounds [0.01, 10] × [0.01, 10], $\mu =\left(\begin{array}{l} 5\\ 5 \end{array}\right)$, and $\sum =\left(\begin{array}{ll} 0.7 & 0.1\\ 0.1 & 0.55 \end{array}\right)$.

**Figure 6.**
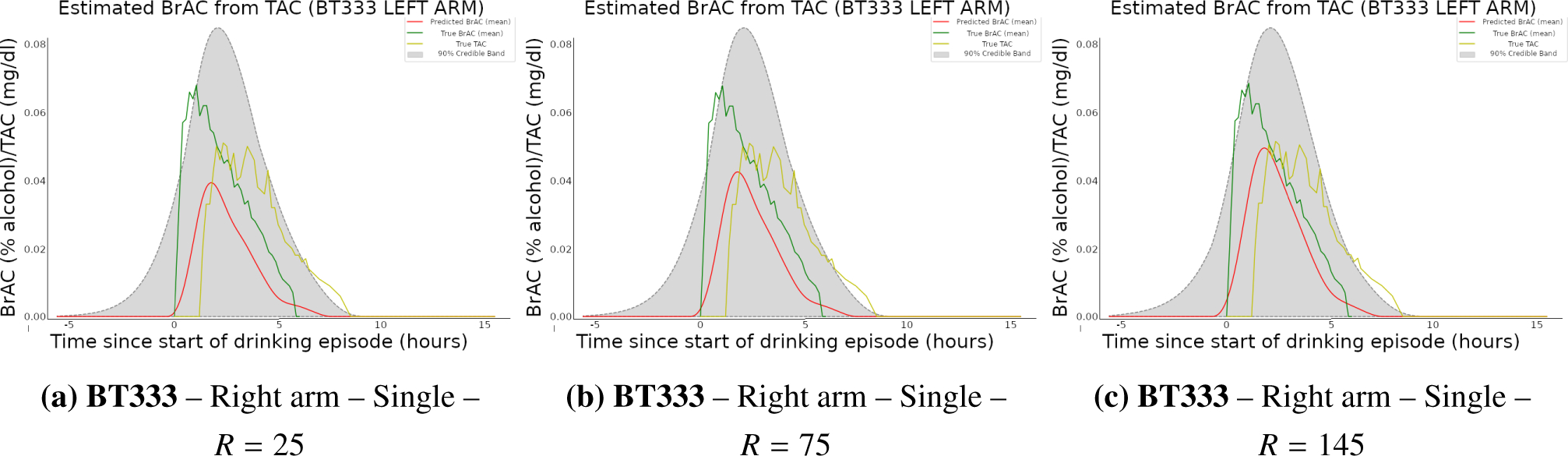
BrAC deconvolution given TAC and predicted ***q*** values for a single test data drinking episode derived using varying amounts of training data, *R*, with conservative credible regions shaded in gray. All sub-figures use the same test TAC data from a single right arm session from BT333. Prior used was tMVN with bounds [0.01, 10] × [0.01, 10], $\mu =\left(\begin{array}{l} 5\\ 5 \end{array}\right)$, and $\sum =\left(\begin{array}{ll} 0.7 & 0.1\\ 0.1 & 0.55 \end{array}\right)$. Associated data are contained in Table 4.

By linearly interpolating the BrAC and TAC data for each subject in all test and training datasets, we are able to re-sample our data with sampling interval *τ* = 45 seconds, and the time discretization *m* = 1300 previously mentioned. The associated participant IDs, TAC device placement (left vs. right arm), type of drinking pattern used (single, dual, or steady), and number of subjects used in posterior distribution determination (*R*) are labeled in Figures 5 and 6. As in eq. (3.14), we utilized all available non-test subject drinking episode measurements (*R* = 136) to determine population parameters $\left(r_{1}^{*},r_{2}^{*}\right)$ to be (4.7733, 1.7020).

Figure 5, shows varying deconvolution attempts for three test data participants, whereas Figure 6 shows deconvolution attempts for the same test data participant reading (**BT333**), with varying amounts of training subject data within the posterior eq. (2.22), *R* = 25, 75, 145. In both Figures 5 and 6, gray bands represent 90% error regions that are determined by sampling respective parameter posterior distributions and utilizing these samples with (3.13) to determine estimated BrAC values. It follows that these error regions contain the 90% credible regions for the pointwise BrAC values as functions of the population parameters appearing in the model. This is the basis for our referring to them in what follows as *conservative credible bands*.

## 4. Discussion

### 4.1. Bayesian estimation of model parameters

Figure 2 illustrates rapid convergence in dimensionality of our spatial dimensions as *n* grows, thus bolstering the results of Theorem 3.1. Within two steps (*n* = 3), we have a graph that visually differs from that of *n* = 25 in ways barely perceptible. Paired with the credible circles in Table 1, these provide evidence that after *n* = 3 the mean and radius of the ***q*** credible circles stay consistent. Thus one can choose a computationally efficient *n* value that minimizes data lost when projecting eq. (2.6) into finite dimensions, eq. (2.11).

For the consistency results, Table 2 exemplifies the theoretical prediction in Theorem 3.5 that as the amount of subject data *R* grows, the posterior distribution better predicts the true ***q*** value by localizing the true parameter ***q***_0_ in mean with higher confidence (smaller credible circles). This increasing confidence is backed by the decreasing variance results shown in Figure 4. Notice that although the variance decreases, the mean is allowed to shift as more data are incorporated, as evident from comparing Figure 4c to Figure 4d. This shifting mean is permitted by the theoretical results and is likely due to the incorporation of 70 extra data points. Table 3 displays the shifting of the mean as more data are incorporated while quantitatively displaying a decreasing 90% credible circle radius, as expected.

As a final note, recall that TAC data were collected simultaneously from both the right and left arms of participants. For an investigation into this see [32].

### 4.2. Deconvolution of BrAC from TAC

In Figure 5a, the deconvolved mean BrAC curve more closely resembles the overall curve of the measured TAC values rather than the desired BrAC, with its increased values towards the latter part of the curve. This is to be expected as the measured TAC plays a role in the Bayesian step, but notice that the severity of the increase in the mean value curve is attenuated when compared to that of the TAC curve (red vs. yellow curves at the five hour mark). A similar phenomenon also appears in Figure 6a. For Figures 6a to 6c, as the number of subject drinking episodes *R* increases, we find that the mean curve grows towards the actual BrAC curve, an expected convergence phenomenon given the theoretical consistency results from Section 3.2.

Lastly, the 90% conservative credible bands about the deconvolved BrAC curves appear to always have a lower bound of zero. For the upper bound, the extreme case is shown in Figure 5c. These wide ranges in BrAC values allow us to capture the true BrAC value with high probability, but also leave us capturing far more area under the curve than needed. Thus, there are times when our two-step method would falsely signal that the TAC device wearer is far more inebriated than they actually are. This incorrect signaling might be due in part to the quantitative inaccurate readings in Figure 5c, wherein the TAC curve is greater than the BrAC curve. If our (training) data are mainly composed of the other cases (TAC following BrAC at an attenuated rate), then the algorithm will learn to “guess up” when turning the TAC back into BrAC. Lastly, this phenomenon may be due to the use of an uninformed prior as the credible regions in Table 3 do not approach zero. Hence, in the future use of an informed prior may be preferable.

### 4.3. Concluding remarks

We believe that the i.n.i.d. assumption from Section 2.2 (specifically Section 3.2) may not reflect the realities of the data collection method wherein two sensors are worn simultaneously on participants’ left and right arms. We are currently investigating the elimination of this i.n.i.d assumption. However, the results from Section 3.4 are quite reasonable and are extremely useful when seeking to use this approach computationally in practice. Further investigation is needed regarding the traveling mean exhibited in the numerical results and how it is related to the non-inclusion of other covariate data (age, height, weight, etc.). This investigation may also be aided by attempting to combine the results of Sections 3.1 and 3.2 and let both the approximating dimension of the kernel, as well as the amount of training data, go to infinity simultaneously.

We also believe that the packaging of all error sources into a single random variable in Section 2.2 may yield larger uncertainties than formulations where many additive errors are considered. Namely, mixed-effects formulations may be utilized in order to separate errors and might lower overall uncertainty. However, the results from Section 3.4 are again quite reasonable, and the usage of mixed-effects formulations can be left as a design choice when considering the main goals and implementations of the PDE model from Section 2.1.

When our approach and results are optimized for use in actual practice, some care will have to be taken in regard to the sampling methods used in Sections 3.4.1 and 3.4.2. If Markov Chain Monte Carlo methods are still the method of choice, then issues such as sample size, convergence of the chains, and randomized chain starting points will need to be taken into account. In addition, a laboratory protocol will be need to be developed to estimate the sensor-dependent values of *q*_3_ and *q*_4_ that appear in eq. (2.3). As far as the numerical results presented in Section 3.4 are concerned in regard to the values chosen for *q*_3_ and *q*_4_, they primarily serve to reinforce the theoretical results in Sections 3.1 and 3.2.

Finally, Of primary interest is the direct inversion of BrAC, *u*, given TAC as in eq. (2.12) without the need for a two-step process like that of the method used in this paper. We believe that a hierarchical model paired with a Gaussian Process framework may reduce the problem down to a single step (see, [52]). In such a framework, we place a prior on ***q***, as well as a function space prior over *u*. In this way, we obtain a method that statistically deconvolves BrAC from TAC while providing a distribution from which we may derive error bars on the estimated BrAC values. We are also currently examining the inclusion of another hierarchical Bayesian model that incorporates covariates in both priors placed over ***q*** and *u*. We believe that this will improve the accuracy of our predictions by allowing the use of all available subject and environment data.

## Figures and Tables

**Table 1.** 90% credible circles for MCMC sampled posteriors with noise distribution $N\left(0,0.005^{2}\right)$.

*n* Dimension	1	2	3	25
Mean (*q*_1_, *q*_2_)	(0.7185, 0.8512)	(0.6829, 0.8651)	(0.6776, 0.8686)	(0.6719, 0.8716)
Credible Circle Radius	0.1173	0.1097	0.1029	0.1289

**Table 2.** 90% credible circles for MCMC sampled posteriors from idealized TAC data with noise distributed $N\left(0,0.025^{2}\right)$ and prior as determined in Section 3.4.2.

*R*	1	11	26	101	276
Mean (*q*_1_, *q*_2_)	(0.683, 1.023)	(0.734, 1.031)	(0.776, 1.025)	(0.877, 1.011)	(0.942, 1.003)
Cred. Circle Radius	0.2854	0.1963	0.1677	0.1590	0.0787

**Table 3.** 90% credible circles for MCMC sampled posteriors with noise distributed $N\left(0,0.025^{2}\right)$ and prior distribution equivalent to the one in Section 6 of [24], namely a tMVN with $\mu =\left(\begin{array}{c} 0.6318\\ 1.0295 \end{array}\right)$, $\Sigma =\left(\begin{array}{cc} 0.0259 & 0.0077\\ 0.0077 & 0.1232 \end{array}\right)$, and ***q*** bounds [0.01, 2.2877] and [0.01, 2.1410].

*R*	1	11	26	101
Mean (*q*_1_, *q*_2_)	(0.913, 1.251)	(1.426, 1.629)	(1.900, 1.551)	(2.183, 1.231)
Credible Circle Radius	0.2824	0.1497	0.1560	0.1254

**Table 4.** Data associated with posterior determination and deconvolution used in Figure 6.

Arm	*R*	*q* mean	90.0% Credible Circle Radius	*q*_1_ range	*q*_2_ range

Right	25	[4.512, 1.346]	1.431	[3.082, 5.943]	[0.01, 2.777]
Right	75	[3.450, 1.215]	1.490	[2.006, 4.986]	[0.01, 2.705]
Right	145	[2.824, 1.000]	1.066	[1.758, 3.890]	[0.01, 2.066]
